# Marine bacteriophages disturb the associated microbiota of *Aurelia aurita* with a recoverable effect on host morphology

**DOI:** 10.3389/fmicb.2024.1356337

**Published:** 2024-03-11

**Authors:** Melissa Stante, Nancy Weiland-Bräuer, Avril Jean Elisabeth von Hoyningen-Huene, Ruth Anne Schmitz

**Affiliations:** Institute of General Microbiology, Christian-Albrechts University Kiel, Kiel, Germany

**Keywords:** metaorganism, *Aurelia aurita*, bacteriophage, microbiome, host–microbe interaction, dysbiosis, fitness

## Abstract

The concept of the metaorganism describes a multicellular host and its diverse microbial community, which form one biological unit with a combined genetic repertoire that significantly influences health and survival of the host. The present study delved into the emerging field of bacteriophage research within metaorganisms, focusing on the moon jellyfish *Aurelia aurita* as a model organism. The previously isolated *Pseudomonas* phage BSwM KMM1 and *Citrobacter* phages BSwM KMM2 – KMM4 demonstrated potent infectivity on bacteria present in the *A. aurita*-associated microbiota. In a host-fitness experiment, Baltic Sea subpopulation polyps were exposed to individual phages and a phage cocktail, monitoring polyp survival and morphology, as well as microbiome changes. The following effects were obtained. First, phage exposure in general led to recoverable malformations in polyps without affecting their survival. Second, analyses of the community structure, using 16S rRNA amplicon sequencing, revealed alterations in the associated microbial community in response to phage exposure. Third, the native microbiota is dominated by an uncultured likely novel *Mycoplasma* species, potentially specific to *A. aurita*. Notably, this main colonizer showed resilience through the recovery after initial declines, which aligned with abundance changes in Bacteroidota and Proteobacteria, suggesting a dynamic and adaptable microbial community. Overall, this study demonstrates the resilience of the *A. aurita* metaorganism facing phage-induced perturbations, emphasizing the importance of understanding host-phage interactions in metaorganism biology. These findings have implications for ecological adaptation and conservation in the rapidly changing marine environment, particularly regarding the regulation of blooming species and the health of marine ecosystems during ongoing environmental changes.

## 1 Introduction

A metaorganism, as defined by Bosch and McFall-Ngai (2011), encompasses the intricate interplay between a multicellular host and its diverse community of microorganisms (Bosch and McFall-Ngai, 2011; Jaspers et al., 2019). By contributing their unique genetic diversity, all members of the community cooperate to ensure the health and survival of the host. Metaorganism research seeks to unravel the intricate host-microorganism interactions and their consequences for the metaorganism’s well-being (Mirzaei and Maurice, 2017; Griem-Krey et al., 2023; Masanja et al., 2023; Shahpari, 2023). Among these complex relationships, the role of bacteriophages is emerging as a compelling field of investigation. Bacteriophages, or phages for short, play crucial roles in marine environments, particularly within the context of metaorganisms (Bosch and McFall-Ngai, 2011; Salmond and Fineran, 2015; Deines et al., 2017). Phages are abundant and highly diverse in marine environments. They are natural predators of bacteria, infecting and lysing bacterial cells during their lytic life cycle (Abedon, 2008; Hobbs and Abedon, 2016). This predation plays a role in controlling and regulating bacterial populations in the marine ecosystem. Thus, phages influence the structure and dynamics of bacterial communities, also affecting the associated microbiota of metaorganisms (Moebus and Nattkemper, 1981; Martha et al., 2011; Koskella and Meaden, 2013). Phage-mediated bacterial lysis releases nutrients and organic matter into the environment and thus contributes to the turnover of organic material, which can be essential for the health and nutrient supply of the host (Martha et al., 2011; Weitz et al., 2015). Phages are often highly specific to their bacterial hosts due to specific receptors on the bacterial cell surfaces. As a result, they can selectively target and infect certain bacterial strains (Culot et al., 2019; Hyman, 2019). This specificity can lead to shifts in bacterial community composition due to phage challenge, favoring specific bacterial taxa while others are suppressed (Middelboe et al., 2001; Pereira et al., 2011). The resulting changes in the microbiota can directly affect the host’s health. Moreover, genetic material can be exchanged between the phage and the host bacterium during phage infection (Thompson et al., 2011; Kirsch et al., 2021). This transduction process can lead to genetically-modified bacterial populations potentially influencing the metaorganism’s adaptation and evolution (Bang et al., 2018; Henry et al., 2021; Voolstra et al., 2021). Bacteriophages further contribute to ecological resilience in marine environments. They support maintaining diversity among bacterial populations by preventing bacterial strains from dominating (Koskella and Brockhurst, 2014; Garin-Fernandez et al., 2018; Batinovic et al., 2019). This bacterial diversity is essential for the stability and functioning of marine ecosystems and the health of metaorganisms (McFall-Ngai, 2014; Wong et al., 2016; Deines et al., 2017; Esser et al., 2019).

*Aurelia aurita*, commonly known as the moon jellyfish, is an excellent model for advancing our understanding of metaorganism biology including phage effects. *Aurelia aurita* belongs to the phylum Cnidaria and is considered a basal metazoan (Matveev et al., 2012). As one of the earliest branching metazoan lineages, it offers unique insights into the evolution of host-microorganism interactions. *Aurelia aurita* has a relatively simple body plan, making it an accessible model organism for such research (Weiland-Bräuer et al., 2015, 2020a,b; Jensen et al., 2023). The ecological relevance of these jellyfish is substantial, as they are widespread in coastal and open-ocean environments, sometimes with detrimental consequences for marine ecosystems, fisheries, industry, and tourism (Dong, 2019; Goldstein and Steiner, 2020). This combination of simplicity and ecological importance makes *A. aurita* an ideal candidate for studying the basic principles of metaorganism interactions in a real-world context. Furthermore, *A. aurita* harbors a diverse microbiota. Extensive research efforts have been dedicated to characterizing the microbiota associated with *A. aurita* and its role in the metaorganism (Weiland-Bräuer et al., 2015, 2020a,b; Jensen et al., 2023). These studies have unveiled the presence of diverse bacterial taxa representing a variety of taxonomic groups, including Proteobacteria, Actinomycetota, Bacilli, and Flavobacteriia (Weiland-Bräuer et al., 2015). These community patterns change with compartment, provenance, and within *A. aurita*’s complex life cycle (Weiland-Bräuer et al., 2015). In the absence of the natural microbiome, a critical asexual reproduction mechanism in the moon jellyfish is severely compromised (Weiland-Bräuer et al., 2020a,b; Jensen et al., 2023). Moreover, health, growth, and feeding rates were decreased in the absence of and upon changes in the community compositions of the native microbiota, e.g., when challenged with pathogenic bacteria (Weiland-Bräuer et al., 2020a,b). While the significance of phages in microbial communities is acknowledged, their role within metaorganisms remains largely unexplored (Nilsson, 2014; Salmond and Fineran, 2015; Lomelí-Ortega et al., 2022). Recently, four virulent phages targeting bacteria present in the *A. aurita*-associated microbiota were isolated from the Baltic Sea, one *Pseudomonas* phage BSwM KMM1 and three *Citrobacter* phages BSwM KMM2 - BSwM KMM4 (Stante et al., 2023). All isolated phages displayed virulent characteristics, including high adsorption rates to the respective bacterial host cells, short latent periods, large burst sizes, and high plating efficiency, signifying their potency and infectivity (Hyman, 2019; Stante et al., 2023). Notably, phages KMM2-4 infected representatives of *Citrobacter*, while KMM1 demonstrated a broad host range, infecting Gram-negative *Pseudomonas* and Gram-positive *Staphylococcus* (Stante et al., 2023). The isolation of these phages opened up further research approaches, including manipulating the *A. aurita*-associated microbiota and gaining insights into the impact of phages on the multicellular host and the metaorganism as a whole. Consequently, we aimed to investigate the complex interactions between microbial communities of the *A. aurita* metaorganism and bacteriophages but also shed light on understanding the dynamic and adaptable nature of a marine host. We conducted a host-fitness experiment with the benthic life stage polyp of the Baltic Sea subpopulation of *A. aurita*. Polyps were challenged with the previously isolated phages KMM1-4 and with a cocktail of all phages to determine changes in the microbial community associated to the *A. aurita* polyps. Moreover, the effects of a disturbed microbiome on survival and polyp morphology were monitored. We ultimately aimed to gain insights into the complexity of host-bacteria-phage interactions.

## 2 Materials and methods

### 2.1 *Aurelia aurita* polyp husbandry, production of sterile polyps, and generation of the native microbiota

*Aurelia aurita* polyps of the Baltic Sea subpopulation were maintained at 20°C in artificial seawater (ASW; 18 practical salinity units (PSU); Tropical Marine Salts, Tropic Marin) in 2 L plastic tanks. Polyps were fed twice weekly with freshly hatched *Artemia salina* (HOBBY, Grafschaft-Gelsdorf, Germany) and washed weekly with ASW. Before the experiment and generation of sterile polyps, animals were not fed for at least 3 days to ensure empty guts (for details, see Weiland-Bräuer et al., 2020a,b; Jensen et al., 2023).

Sterile polyps were generated using an established broad-spectrum antibiotic mixture (50 mg/L of chloramphenicol, neomycin, ampicillin, streptomycin, rifampicin, and 60 mg/L of spectinomycin; Carl Roth, Karlsruhe, Germany) as described by Jensen et al. (2023), including antimycotics (3.5 mg/L of neomycin, and amphotericin; Carl Roth, Karlsruhe, Germany). The antibiotic mixture was prepared using sterile ASW (filtered through a 0.22 μm polycarbonate syringe filter; Sartorius, Goettingen). Polyps were kept in the antimicrobial mixture for 3 days, which was refreshed twice daily. Germ-free polyps were confirmed by testing for the absence of bacterial 16S rRNA gene products through amplification within a standard PCR using the primer set 27F and 1492R (Zavaleta et al., 1996). DNA extraction and testing were performed on randomly selected polyps (6 replicates) using the WIZARD Genomic DNA Purification kit (Promega GmbH, Walldorf, Germany), according to the manufacturer protocol (Weiland-Bräuer et al., 2020a,b; Jensen et al., 2023).

The generation of the *A. aurita*-associated native microbiota is described in detail in (Jensen et al., 2023). In short, the *A. aurita*-associated native microbiota was generated by collecting 20 native polyps, washing them with sterile artificial seawater (ASW) to eliminate transient bacteria, and transferring them into 500 μL fresh sterile ASW. Mechanical homogenization and filtration were performed to remove eukaryotic cells. Bacterial cell numbers were determined from fluorescently-labeled cells (1:1000 SYTO9; Invitrogen, Darmstadt, Germany) with a Neubauer count chamber (Assistant, Sondheim vor der Röhn, Germany; Axio Scope microscope and Axio Vision software, Zeiss, Jena, Germany) according to the manufacturer’s instructions. The absolute number of bacterial cells was determined with 3.6 × 10^6^ cells/polyp (Jensen et al., 2023).

### 2.2 Phage lysate preparation

*Pseudomonas* phage BSwM KMM1 (KMM1), *Citrobacter* phage BSwM KMM2 (KMM2), *Citrobacter* phage BSwS KMM3 (KMM3), and *Citrobacter* phage BSwM KMM4 (KMM4) were previously isolated from Baltic Sea water (Stante et al., 2023). The phages were shown to infect *A. aurita* bacterial colonizers *Pseudomonas* sp. (accession no. MK967010.1, primary target host of KMM1), *C. freundii* (accession no. OQ398153, primary target host of KMM2), and *Citrobacter* sp. (accession no. OQ398154, primary target host of KMM3 and KMM4). For phage lysate preparation, the bacterial strains were grown in 5 mL Marine Bouillon (MB, 10 g/L yeast extract, 10 g/L peptone (Carl Roth, Karlsruhe, Germany); 30 PSU Tropical Marine Salts; pH 7.3) overnight at 30°C and 120 rpm. 48 mL MB medium in a 100 mL Erlenmeyer flask was inoculated with 1 mL of each overnight culture and incubated at 30°C and 120 rpm. Once the optical density at 600 nm reached 0.2–0.3. 1 mL, phages were added to the cultures at a concentration of 10^6^ pfu/ml, which were further incubated for 3 h at 30°C and 120 rpm. The cultures were transferred to 50 mL Falcon tubes (Sarstedt, Nümbrecht, Germany) and centrifuged at 4000 ×*g* for 10 min. The supernatant was sterile-filtered using a 0.22 μm filter (Sartorius, Goettingen, Germany). The concentration (pfu/ml) of the resulting phage lysate was determined using the double agar layer technique (Cormier and Janes, 2014; Abedon, 2021). Subsequently, 50 mL of freshly prepared phage lysate (> 10^8^ pfu/mL) were ultracentrifuged (Optima XE-100 ultracentrifuge, Beckman Coulter, Brea, CA, United States) at 109,800 × *g* for 30 min. Phage pellets were resuspended in 1 mL of Ultra-pure water (Carl Roth, Karlsruhe, Germany) overnight at 4°C on a 3D shaker and stored at 4°C.

### 2.3 Monitoring phage-induced malformation of *Aurelia aurita* polyps

**Figure 1:**
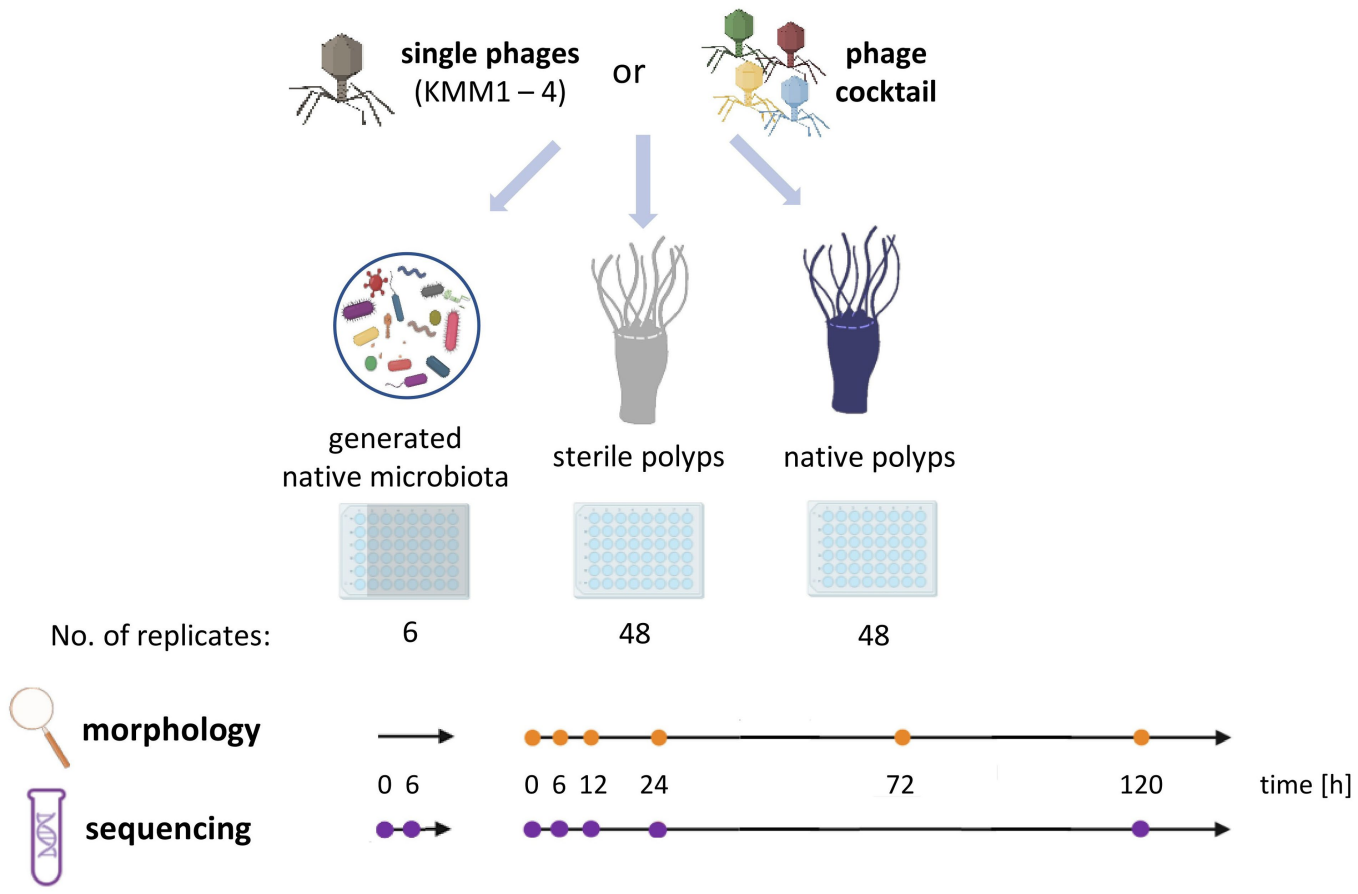
Experimental design of the host-fitness experiment. Single phages KMM1 – KMM4 (1 × 10^6^ pfu/polyp) and a phage cocktail consisting of equimolar amounts of all phages (4 × 10^6^ pfu/polyp) were added to the generated native microbiota of *Aurelia aurita* polyps as well as to sterile and native polyps. Polyps were phenotypically analyzed over 120 h at the specified time points (orange dots) using a stereomicroscope (Novex Binocular RZB-PL Zoom-Microscope 65.500, Arnhem, Netherlands). Microbiome analyses were conducted at the indicated time points (violet dots) using 16S rRNA amplicon sequencing.

Phage-induced changes in survival rates and related malformations of *A. aurita* polyps were observed to evaluate the fitness of polyps. For this purpose, single native and sterile polyps of the Baltic Sea subpopulation (48 replicates each) were transferred from 2 L husbandry tanks to 48-well plates with 1 mL native or sterile 18 PSU ASW per well. Six different conditions (untreated vs. phage-treated) were used to study the impact of phages on the *A. aurita*-associated microbiota and, consequently, on the host’s fitness (Figure 1). In more detail, single *A. aurita* polyps (bacterial cells 3.6 × 10^6^ cells/polyp) were incubated in 1 mL ASW with 10 μL of 1 × 10^8^ pfu/ml of each of the phages KMM1 – KMM4 separately, and the phage cocktail (4 × 10^8^ pfu/ml) for up to 120 h. Phage titers were randomly controlled for both polyps and ASW after 120 h with the double agar layer technique (Cormier and Janes, 2014; Abedon, 2021). Polyps were monitored with a stereomicroscope (Novex 173 Binokulares RZB-PL Zoom-Mikroskop 65,500, Novex, Arnhem, The Netherlands) integrated with an HDMI/HD camera at 0 h and after 6, 12, 24, 72, and 120 h. The fitness of polyps was categorized into unaffected, malformed, and dead according to their phenotypic appearance, dimensions, and presence of tentacles. Phage-induced effects on the polyp morphology were compared between control polyps (native, untreated) before the addition of phages (0 h) and phage-treated polyps at each observed time point (6, 12, 24, 72, and 120 h).

In parallel, 16S rRNA amplicon sequencing was performed with six randomly selected native polyps (parallel set up of an additional 48-well plate with 48 replicates) to analyze potential changes in the microbial community composition based on the phage treatments, and ultimately correlating observed effects on the polyp morphology with changes in microbial community patterns. Native polyps were removed for DNA isolation with the WIZARD Genomic DNA Purification kit (Promega GmbH, Walldorf, Germany), according to the manufacturer’s instructions and subsequent 16S rRNA amplicon sequencing before the start of the experiment (0 h) and after 6, 12, 24, and 120 h.

In addition, the impact of phages on the native microbiota of polyps alone was evaluated in absence of the host. For this, six replicates of the generated microbiota (each with 3.6 × 10^6^ cells/ml) were incubated in 1 mL ASW with 10 μL (1 × 10^8^ pfu/ml) of each phage (KMM1–4) and the phage cocktail (4 × 10^8^ pfu/ml) for 6 h. The uninfected generated microbiota sampled after 6 h of incubation acted as the control. According to the manufacturer’s instructions, DNA was isolated from the collected samples with the WIZARD Genomic DNA Purification kit (Promega GmbH, Walldorf, Germany), and subsequent 16S rRNA amplicon sequencing was performed.

### 2.4 Bacterial 16S rRNA amplicon sequencing and downstream analysis

The microbial community composition of native control polyps (untreated), phage-treated polyps, and the generated native microbiota were investigated using 16S rRNA amplicon sequencing. Six polyps (control, phage-treated) were randomly removed from 48-well plates at 0 h and after 6, 12, 24, and 120 h. The generated native microbiota was analyzed at 0 h and after 6 h. The DNA was isolated using the WIZARD Genomic DNA Purification kit (Promega GmbH, Walldorf, Germany) according to the manufacturer’s instructions. The hypervariable regions V1-V2 of the bacterial 16S ribosomal RNA gene were amplified in a dual-barcode approach from the extracted DNA using the forward primer V2_A_Pyro_27F5′-CGTATCGCCTCCCTCGCGCCATCAGTCAGAGTTTGATCCTGGCTCAG-3′ and the bar-coded reverse primers V2_B_338R5′-CTATGCGCCTTGCCAGCCCGCTCAGCATGCTGCCTCCCGTAGGAGT-3′ (Caporaso et al., 2012). The amplicon libraries were constructed and sequenced on an Illumina MiSeq v3 platform (2 × 300-cycle kit) at the Competence Centre for Genomic Analysis (Kiel, Germany). Sequence data were provided as demultiplexed sequences with quality profiles generated using FastQC (Andrews, 2010). Sequences were quality filtered using fastp (Chen et al., 2018) to remove sequencing adapters. Reads shorter than 50 bp and a Phred score below 20 were removed. All subsequent steps used the Qiime2 framework (Bolyen et al., 2019). In short, quality-filtered sequences were imported into Qiime2 and truncated at 285 bp for forward and 240 bp for reverse reads before denoising. Each sequencing run was separately denoised and clustered into amplicon sequence variants (ASVs) using the dada2 plugin (Callahan et al., 2016). The denoised data were then merged using the feature-table merge and merge-seqs commands. Dereplicated and merged representative ASVs were taxonomically assigned using the pre-formated SILVA 138 SSURef NR99 full-length database (Gurevich et al., 2013), prepared using the RESCRIPt plugin (Robeson et al., 2021). Taxonomic assignment was done using the Qiime2 feature classifier (Bokulich et al., 2018) with the vsearch plugin (Rognes et al., 2016) and default settings. Amplicon sequence data were deposited under the NCBI BioProject PRJNA1010146, and BioSample Accessions SAMN37180193-SAMN37180465.

A phylogenetic tree was calculated based on the merged dereplicated ASV sequences. For this purpose, the sequences were aligned with mafft (Katoh et al., 2002), and a maximum-likelihood tree was calculated using FastTree (Price et al., 2010). The ASV table, midpoint-rooted phylogenetic tree, metadata and dereplicated reference sequences were exported using Qiime tools (Bolyen et al., 2019) and biom tools (McDonald et al., 2012).

### 2.5 Data analysis and statistics

Amplicons were analyzed using RStudio (2022.07.0 + 548) and R version 4.2.3. The data were managed and reformatted using the packages phyloseq (McMurdie and Holmes, 2013), microViz (Barnett et al., 2021), and ampvis2 (Andersen et al., 2018). Contaminant amplicon sequences were identified and removed with decontam (Davis et al., 2018) using prevalence-based identification with a prevalence threshold of 0.5 and negative sequencing controls as a reference. Unwanted sequences, such as archaea, chloroplasts, and mitochondria, were excluded from the ASV table in a further filtering step. The filtered ASV table was normalized using DeSeq2 (Love et al., 2014) and transformed into relative abundances for visualization. Principal coordinates analyses (PCoA) were generated using weighted UniFrac distance matrices based on all ASVs with a relative abundance above 0.01% and the ampvis2 package (Andersen et al., 2018). Barcharts of relative abundances were plotted in Excel.

To provide a general overview of the changes among principal ASVs on the microbiota of polyps after each treatment, a heatmap was created using CLUSTVIS (Metsalu and Vilo, 2015).^1^ Considering the median values of the relative abundance of each treatment at different time points, values >0.1% were utilized for the calculations. The heatmap plot represents the relative proportion of each ASV (Y-axis, ordered by phylum in descending order) within each treatment at different time points (X-axis). Changes in values were standardized against control polyps by comparing the median values derived from six replicates for each treatment at different time points with those of control polyps at 0 h.

For the calculation of diversity and richness indices, the unnormalized ASV table was rarefied to 10,000 reads. Shannon (H′) diversity index was calculated using ampvis2 and vegan 2.6–4 (Oksanen et al., 2022) Faith’s phylogenetic diversity (Faith’s PD) was calculated using the picante package and the mid-point rooted phylogenetic tree (Kembel et al., 2010). Differences in diversity between treatments and timepoints were tested for statistical significance using paired Wilcoxon (Kruskal, 1957) and Kruskal–Wallis tests (William and Allen, 1952). The significance of morphology effects due to phage treatments at each timepoint was tested using a proportionality test (2-proportions test without ‘Yates’ continuity correction) with a significance cut-off of *p* ≤ 0.05 and the stats package 4.2.3 in R (Supplementary Table S1).

### 2.6 Phylogenetic analysis of the *Aurelia aurita*-specific uncultured *Mycoplasma*

In order to determine the phylogenetic assignment of the highly abundant yet uncultured *Mycoplasma* strain, the genomic DNA of a pool of 10 Baltic Sea *A. aurita* polyps was isolated using the Wizard Genomic DNA Purification Kit (Promega GmbH, Walldorf, Germany) according to the manufacturer’s instructions. The full-length bacterial 16S rRNA gene was PCR-amplified from 50 ng of isolated genomic DNA using the bacterium-specific 16S rRNA gene primer 27F (5′-AGAGTTTGATCCTGGCTCAG-3′) and the universal primer 1492R (5′-GGTTACCTTGTTACGACTT-3′) (Zavaleta et al., 1996), resulting in a 1.5 kb PCR fragment. The PCR product was purified using Macherey-Nagel™ NucleoSpin™ Gel and PCR Clean-up XS kit (Thermo Fisher Scientific, Hessen, Germany). The ligation of the purified PCR product was performed using Invitrogen™ TOPO™ TA Cloning™ Kit, Dual Promoter (Thermo Fisher Scientific, Hessen, Germany), following the manufacturer’s instruction. After the blue-white screening, 24 white insert-containing colonies were selected for plasmid purification using the Presto™ Mini Plasmid Kit (PDH300) (Geneaid Biotech Ltd., New Taipei City, Taiwan) according to the company’s guidelines. Inserts were Sanger-sequenced by the Institute of Clinical Molecular Biology in Kiel, Germany. Sequence analysis (quality check, assembly of forward and reverse reads) was conducted using Geneious Primer (v2022.02) resulting in a sequence with 1,283 bp. Sequence data of the near full-length 16S rRNA gene was deposited under the NCBI BioProject PRJNA1010146, and GenBank Accession no. OR634772. Phylogenetic assignment was conducted using the “All Species Living Tree (LTP) project” (release LTP_06_2022)^2^ (Ludwig et al., 2021). The taxonomy was updated as recommended by the documentation of the release. Selected host-associated and endosymbiotic *Mycoplasma* sequences were downloaded from the NCBI database. Together with the *A. aurita*-specific *Mycoplasma* sequence, they were integrated into the LTP tree using the ARB software environment (version arb-7.0) (Ludwig et al., 2004) and the tutorials provided in the documentation (available at: http://www.arb-home.de/documentation.html). The integrated fast aligner was used to align all additional *Mycoplasma* sequences with the ten nearest neighbors within the database. The alignments were manually curated and saved to the database. Sequences were then added to the LTP-tree using the ARB parsimony function. A subset of the LTP-tree was calculated using selected *Mycoplasma* sequences and an *Escherichia coli* outgroup. The subset-tree was built using the neighbor-joining algorithm and 1,000 bootstraps. The phylogenetic tree was exported into newick format and visualized using the interactive Tree Of Life (iTOL) v6 (Letunic and Bork, 2021).

## 3 Results

A host-fitness experiment was conducted using *A. aurita* Baltic Sea polyps. The polyps were exposed to either one of the previously isolated phages BSwM KMM1-4 or the phage cocktail, comprising the four phages in equimolar proportions. The impact of these phages on survival and polyp morphology was assessed, and changes in the microbiome caused by phage-induced disturbance were studied through bacterial 16S rRNA amplicon analyses (Figure 1).

### 3.1 Phage infections with BSwM KMM1-4 caused recoverable polyp malformations

The evaluation of polyp fitness involved monitoring both the survival of polyps and any associated phenotypic changes (Figure 1). Survival of polyps is assessed by monitoring deceased individuals (Supplementary Figure S1A), as these disintegrate after undergoing a notable change in phenotype marked by a roundish, shrunken body (Weiland-Bräuer et al., 2020a,b; Jensen et al., 2023; Pinnow et al., 2023). The morphological assessment further involves categorizing between unaffected and malformed polyps based on their appearance, tentacle presence, and dimensions (Weiland-Bräuer et al., 2020a,b; Pinnow et al., 2023). Unaffected polyps can be identified by a stalk attachment, an elongated calyx, and fully developed, extended tentacles (Supplementary Figure S1B). In contrast, malformed polyps lack at least two of these characteristics, primarily exhibiting a deformed calyx (Supplementary Figure S1Ci) and reduced, absorbed (Supplementary Figure S1Cii), or even absent (Supplementary Figure S1Ciii) tentacles.

Initially, the potential impact of phages on the eukaryotic host was investigated by evaluating polyp survival and phenotypic changes of sterile polyps (Figure 1). Sterile polyps served as the control group, while the experimental group included sterile polyps treated with both individual phages and the phage cocktail. Single sterile polyps were incubated in 48-well plates with 1 × 10^6^ pfu/polyp of one of the phages KMM1–4 and the phage cocktail (4 × 10^6^ pfu/polyp) for up to 120 h. The phage titre was randomly assessed for both polyp and ASW after 120 h, revealing a range of 10^5^–10^6^ pfu/polyp. This indicates that the phages did not propagate, demonstrating their ability to survive for at least 5 days under the experimental conditions. As described in previous studies (Weiland-Bräuer et al., 2020a,b; Jensen et al., 2023), sterile animals were slightly smaller than their native counterparts but exhibited no morphological changes. The phage treatments were survived by all sterile polyps (48 replicates per treatment) without experiencing any alterations in their morphology (Supplementary Figure S2). Thus, the likelihood of direct impact of the phages on the polyp host can be excluded. Consequently, we assume that any effects on native polyps obtained in the main experiment is indirectly caused by phage-induced microbiome changes.

**Figure 2:**
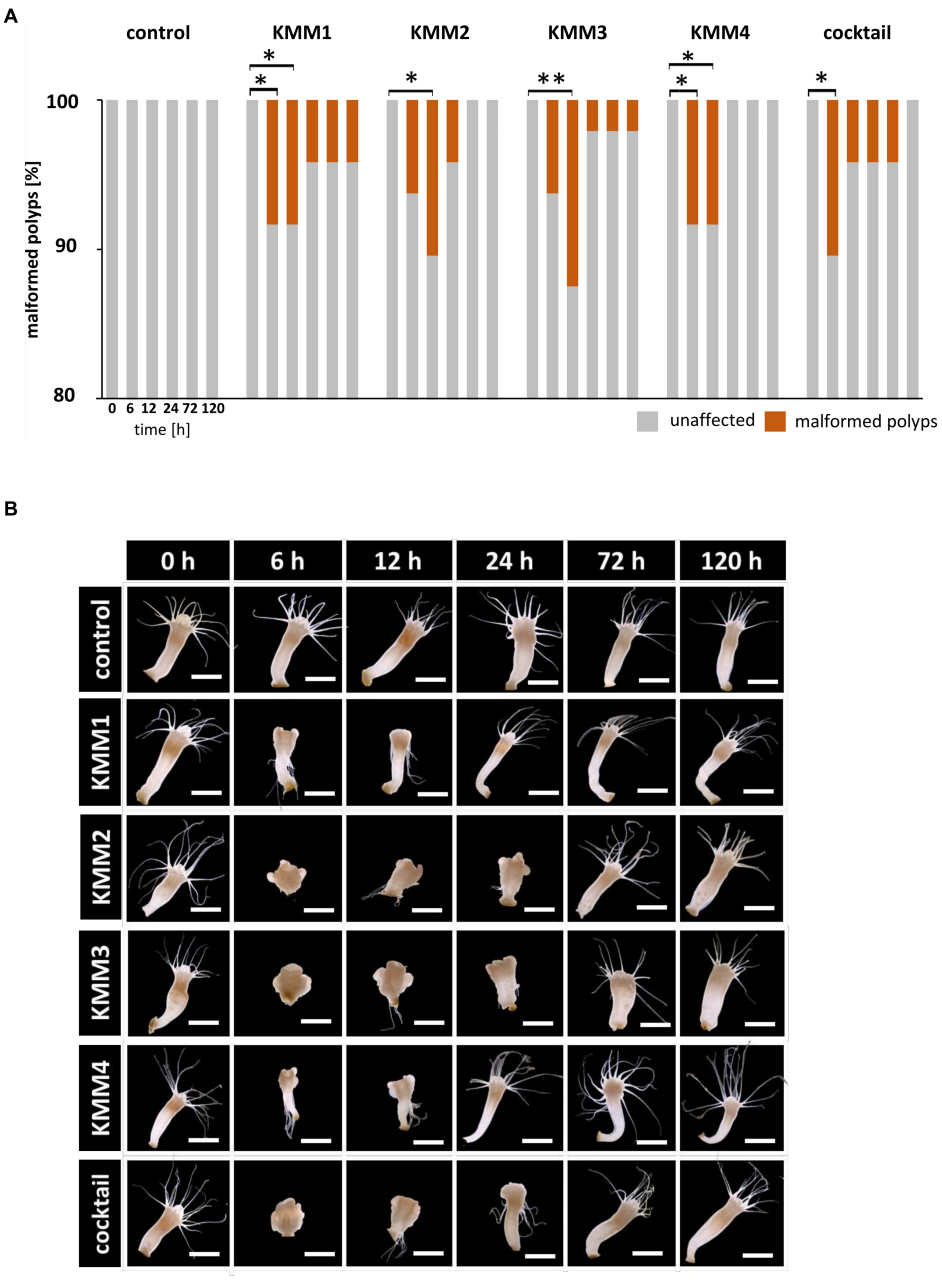
Effect of bacteriophages on the morphology of native *A. aurita* polyps. Native *A. aurita* polyps (48 replicates, each in 1 mL ambient water) were incubated with 10 μL of 1 × 108 pfu/ml of one of the phages KMM1–4, and the phage cocktail (4 × 108 pfu/ml) for up to 120 h. Polyp morphology was monitored, and polyps were categorized into unaffected and malformed. **(A)** Percentage of morphologically altered polyps over time per treatment. ^*^p < 0.05 represents a significant effect **(B)** Representative original photographs per treatment and time points exemplify polyps’ phenotypical appearance (same polyp per treatment) using a stereomicroscope (Novex Binocular RZB-PL Zoom-Microscope 65.500, Arnhem, Netherlands). Scale bars represent 1 mm.

Secondly, the survival of native polyps was examined after 120 h in a similar setup to that of their sterile counterparts (Figure 1) showing that survival rates were unaffected after phage infections (experimental groups) compared to the untreated control (control group), meaning that all 48 replicates survived the single and cocktail phage infections. Subsequently, the polyps’ phenotype was assessed at 0, 6, 12, 24, 72, and 120 h after phage challenges. Only a small number (4–6 polyps, corresponding to 8–17%) of polyps were significantly morphologically affected (*p* < 0.05) by the phage infections (Figure 2A). In more detail, no malformations were observed in control polyps over time (Figure 2A). However, infection with KMM1 led to 8% of polyps showing malformations after 6–12 h (*p* = 0.04; Supplementary Table S1). Interestingly, half of these initially malformed polyps fully recovered after 120 h (Figure 2A). Similar outcomes were obtained for KMM3 infection, with six polyps displaying malformations (*p* = 0.01; Supplementary Table S1), partly recovering between 72 to 120 h (Figure 2A). KMM2 and KMM4 infections also resulted in 8–10% malformed polyps (*p* = 0.02–0.04; Supplementary Table S1), which all recovered 24–120 h post-infection (Figure 2A). Finally, incubation with the phage cocktail lead to five malformed polyps after 6 h, which all recovered over time (Figure 2A). Even though only a small but significant number of polyps displayed phenotypic abnormalities after being exposed to phages (Figure 2A), the magnitude of these deformities was notable. Phenotypic changes were relatively minor when the polyps were infected with KMM1, and slightly stronger with KMM4. They were characterized by deformed calyces and reduced absorbed tentacles, however infections with KMM2, KMM3, and the phage cocktail led to roundish, shrunken body shapes with absorbed tentacles (Figure 2B). The recovery of polyps is particularly noteworthy. The substantial malformations (almost) completely recovered over time (Figure 2B, see 72–120 h, which shows always the same polyp). In conclusion, a significant proportion of *A. aurita* polyps exhibited substantial phenotypic changes in the presence of all phages, likely attributed to alterations in the polyp-associated microbiome. The observed recovery in fitness suggests a fast adaptation of the polyp, potentially facilitated by the rapid re-balancing of its microbiome.

### 3.2 Amplicon sequencing of microbiomes and statistics

Phage-induced changes in the microbiomes of native polyps compared to untreated polyps were analyzed using 16S V1-V2 rRNA amplicon sequencing. In our experimental design, a total of 96 replicates were employed, with 48 allocated for morphological observations and an additional set of 48 earmarked for sequencing purposes. To guarantee a representative sampling during the sequencing phase, six replicates of polyps at various time points (0, 6, 12, 24, and 120 h) were randomly selected. Additionally, the impact of the phages on the generated polyp microbiota in the absence of the host was investigated. Six replicates of native polyps of each treatment at various time points (0, 6, 12, 24, and 120 h) were considered. Amplicon sequencing resulted in 13,708,412 reads, averaging at 32,104 reads per sample. After quality filtering, denoising, merging, and chimera removal, 10,508,909 reads (77%) remained in the dataset.

Clustering into amplicon sequence variants (ASVs) resulted in a total of 3,034 ASVs. Statistical decontamination using decontam resulted in the removal of 21 ASVs identified in the negative controls. After removal of sample duplicates with low read-counts, sequencing controls, as well as ASVs with 0 counts and ASVs assigned to the Archaea, chloroplasts or mitochondria, a total of 296 samples and 2,682 ASVs remained in the dataset (Supplementary Tables S2, S3).

It is noteworthy that this selection process encompassed both malformed and non-malformed polyps, thereby offering a comprehensive representation of the polyp population. Phage-induced changes in the microbiomes of native polyps, as compared to untreated polyps, were analyzed using 16S V1-V2 rRNA amplicon sequencing. Concurrently, we investigated the impact of the phages on the generated polyp microbiota in the absence of the host. The sequencing process resulted in a total of 13,708,412 reads, averaging 32,104 reads per sample. Following quality filtering, denoising, merging, and chimera removal, 10,508,909 reads (77%) remained in the dataset. Clustering into amplicon sequence variants (ASVs) yielded a total of 3,034 ASVs. Statistical decontamination using decontam led to the removal of 21 ASVs identified in the negative controls. Subsequently, after eliminating sample duplicates with low read-counts, sequencing controls, as well as ASVs with 0 counts and ASVs assigned to the Archaea, chloroplasts, or mitochondria, a final dataset of 296 samples and 2,682 ASVs was retained (Supplementary Tables S2, S3).

### 3.3 The generated native microbiota changes after phage infection with KMM3 and KMM4

**Figure 3:**
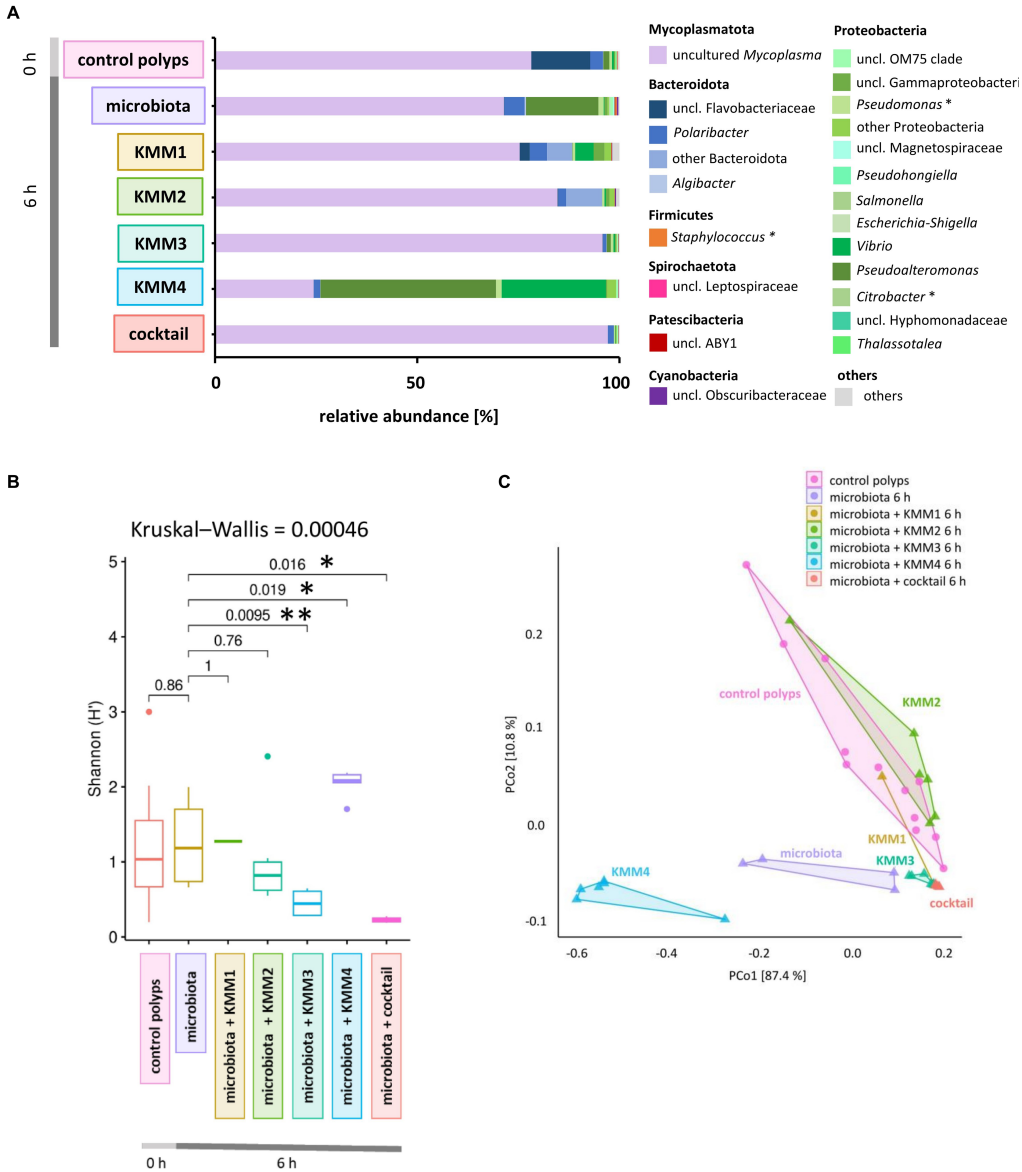
Effect of phage treatments on the generated native *A. aurita* microbiota. The native microbiota was generated from the homogenate of 10 *A. aurita* polyps filtered through a 3.1-mm filter. The native microbiota (3.6 × 10^6^ cells/ml) was incubated in 1 mL with 10 μL of 1 × 10^8^ pfu/ml of one of the phages KMM1–4, and the phage cocktail (4 × 10^8^ pfu/ml) for 6 h at 20°C (each treatment with 6 replicates, in 48-well plates). Microbial communities were analyzed after phage treatment by sequencing the V1-V2 region of the bacterial 16S rRNA gene. **(A)** The bar plot illustrates the composition of the phage-treated generated native microbiota compared to the untreated control. The data was derived from the median values of six biological replicates. Dominant genera (>0.1% relative abundance) are visualized, and genera <0.1% relative abundance are grouped as “others.” Primary phage hosts are denoted with an asterisk*. **(B)** Box plots comparing the Shannon H′ diversity of the bacterial community after phage treatments with the untreated generated microbiota. The global Kruskal–Wallis tests indicates whether overall changes occur in the data, while pairwise Wilcoxon tests indicate conditions with significant differences in diversity (^*^p < 0.05, ^**^p < 0.01). **(C)** The PCoA plot based on the weighted UniFrac distance measure shows the similarity of microbiomes, including data from six replicates per treatment grouped as polygons. Before the analysis, ASVs with a relative abundance of <0.01% in any sample were excluded. The relative contribution (eigenvalue) of each axis to the total inertia in the data is indicated in percent at the axis titles.

The native microbiota associated with *A. aurita* polyps was generated to explore the infection capabilities of the previously identified phages KMM1–4. The generated native microbiota without phages served as the control, while the experimental group comprised the native microbiota in ASW with the addition of phages. Six replicates of the generated microbiota (3.6 × 10^8^ cells/ml) were incubated in 1 mL ASW with 10 μL of phages (KMM1–4) and a phage cocktail (4 × 10^8^ pfu/ml) for 6 h. Prolonged incubation was avoided due to the previously described changes in the microbiota over time in the absence of the host (Jensen et al., 2023). Analysis of 16S rRNA amplicons provided insights into the composition of the untreated generated microbiota, revealing the dominance of uncultured *Mycoplasma* (68%), followed by Proteobacteria (18%) and Bacteroidota (5%) (Figure 3A). A more detailed examination focused on the primary target hosts susceptible to phage infection, revealing a single ASV of *Citrobacter* with a relative abundance of 0.008%. Additionally, three ASVs of *Pseudomonas* and five ASVs of *Staphylococcus* were identified, displaying relative abundances of 1.2 and 0.4%, respectively (Figure 3B). Comparing this microbial community composition with that of native polyps, which served as the source of the generated microbiota, a similar distribution was observed. On native polyps, 76% of the microbiota was composed of uncultured *Mycoplasma*, 18% Bacteroidota, and 3% Proteobacteria (Figure 3A). Shannon indices indicated no significant change in diversity between the samples (Figure 3B). However, alterations in the abundance of Proteobacteria, particularly *Pseudoalteromonas*, were detected in the generated microbiota (Figure 3A). These fluctuations in Proteobacteria abundance in the generated microbiota might be attributed to procedural aspects during microbiota generation, such as polyp homogenization and filtration. The observed variations highlight the importance of considering these procedural nuances when interpreting microbial community dynamics in experimental setups. After phage infection, alpha diversity analysis indicated no noteworthy differences in ASV evenness (Shannon H′) between the untreated and KMM1- and KMM2-treated generated microbiota (Figure 3B). However, when exposed to KMM3, KMM4, and the phage cocktail, substantial changes in diversity were observed compared to the untreated microbiota (*p* < 0.02; Figure 3B). Variations in alpha diversity were attributed to shifts in the abundance of uncultured *Mycoplasma*, Bacteroidota (specifically uncl. Flavobacteriaceae and *Polaribacter*), and Proteobacteria (specifically *Pseudoalteromonas* and *Vibrio*) (Figure 3A). Identified differences in alpha diversity were confirmed in the Principle Coordinate analysis (PCoA) for the infection with KMM3, KMM4, and the cocktail (Figure 3C). In summary, exposure to KMM3, KMM4, and the phage cocktail led to significant changes in the polyp microbiome in the absence of the host. The changes are related to shifts in the abundance of specific microbial taxa, highlighting the potential of these phages to influence the microbiome associated with *A. aurita*.

### 3.4 Microbial community patterns of native polyps change in response to phage treatment

**Figure 4:**
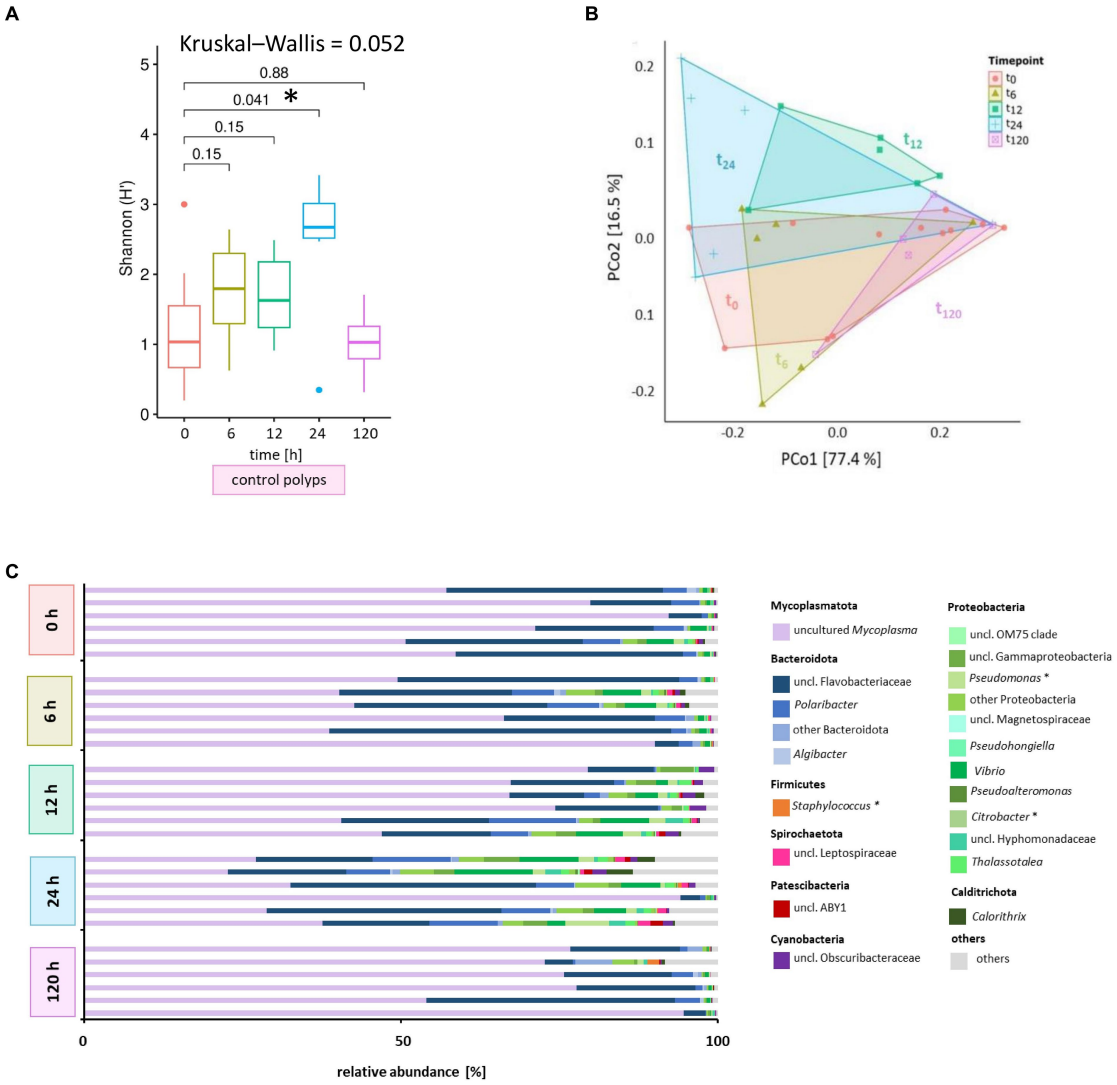
Microbiome analysis of control polyps over time. Bacterial 16S rRNA amplicon sequencing (V1-V2 region) was performed for the associated microbiota of six randomly selected *A. aurita* control polyps (untreated) at five different time points. **(A)** Shannon diversity box plots reflect changes in the alpha diversity metric Shannon H′ for native polyps over time. The results of the pairwise Wilcoxon test are indicated above each pair. Asterisks highlight significant changes with a cut-off of *p* < 0.05. **(B)** Principal coordinates analysis (PCoA) based on the Weighted UniFrac distance visualizes the microbial community’s similarity of untreated polyps over time. Polygons group six polyps per time point. ASVs with a relative abundance <0.01% in any sample were removed before analysis. The percentages of total inertia contributed by each axis are shown in the axis captions. **(C)** Dominant bacterial genera assigned to their phyla are presented as six individually analyzed polyps. The bar plot includes genera reaching at least 0.1% relative abundance. Bacterial genera with <0.1% relative abundance are grouped as others. Primary target hosts of phages KMM1- KMM4 are indicated with an asterisk.

Initially, we examined the potential influence of the incubation period on the microbiome without challenges to monitor the phage impact on the polyp-associated microbiome over 120 h. A subset of *A. aurita* polyps from the Baltic Sea subpopulation (48 replicates) was individually cultivated in 18 PSU ASW (1 mL each). Six native polyps were randomly selected at 0, 6, 12, 24, and 120 h for DNA isolation and subsequent 16S rDNA V1-V2 amplicon sequencing (Figure 1). The alpha diversity metrics of native polyps were generally comparable across most time points, with a statistically significant divergence observed after 24 h (*p* = 0.041) (Figure 4A). The initial microbiome composition (0 h) of native Baltic Sea polyps comprised 76% Mycoplasmatota, 18% Bacteroidota, and 6% other taxa (Figure 3A). The primary target hosts for phage infection had low relative abundances (0.002% *Citrobacter*, 0.5% *Pseudomonas*, 0.02% *Staphylococcus*) (Figure 4C), and this composition remained consistent over 120 h (Figure 4C). Despite marginal alterations in genera abundance, a significant alpha diversity divergence at 24 h was primarily due to changes in evenness, not richness, compared to other samples, with Mycoplasmatota yielding dominance to Bacteroidota and Proteobacteria. PCoA at the ASV level revealed low dissimilarity among replicates and between different time points, with untreated polyps at 0 h used as the comparison control for subsequent analyses (Figure 4B). The Baltic Sea polyps were predominantly colonized by Mycoplasmatota (76%), represented by an uncultured *Mycoplasma* ASV (Figure 4C, violet bars, ASV01479). ASV01479, identified as uncultured *Mycoplasma*, showed high abundance (30–76%) in all Baltic Sea polyp microbiomes. Sanger sequencing and phylogenetic analysis confirmed its affiliation with the genus *Mycoplasma*, potentially representing a novel species exclusive to *A. aurita*. The phylogenetic tree demonstrated clustering with *Spiroplasma*, *Mesoplasma*, *Mycoplasma*, and Candidatus *Marinoplasma*, supporting the hypothesis of a novel *A. aurita*-specific *Mycoplasma* species (Supplementary Figure S3).

Next, a comprehensive analysis comparing untreated control polyps and those subjected to phage exposure was conducted to assess the impact of phage treatments on the microbial community patterns of native polyps, and ultimately correlating the findings with malformation observations (Figure 1). The experimental design involved 48 individual *A. aurita* polyps, divided into a control group (untreated native polyps) and an experimental group (phage-treated native polyps). Polyps were exposed to either phages KMM1–4 or a phage cocktail for up to 120 h, with six replicates randomly selected for subsequent 16S rRNA analysis.

**Figure 5:**
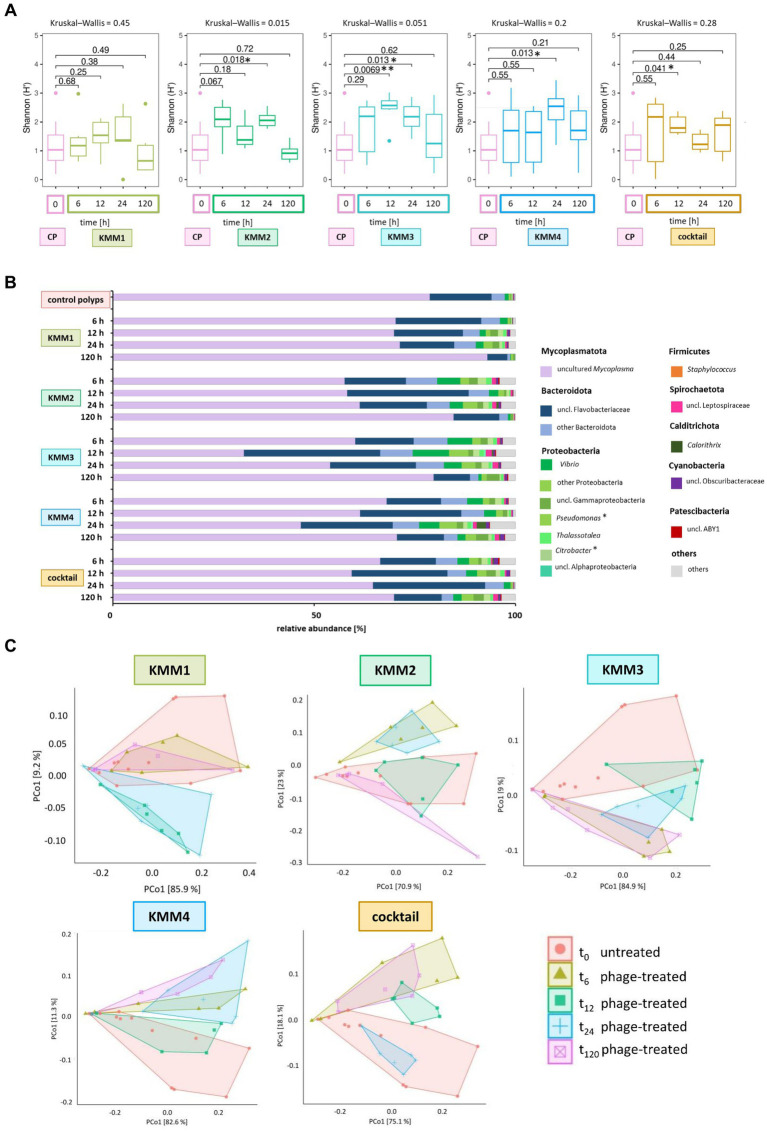
Microbial community changes after KMM1 – KMM4 phage treatment of native *A. aurita* polyps. Individual *A. aurita* polyps (48 replicates, in 1 mL ambient water) were incubated with 10 μL of 1 × 10^8^ pfu/ml of one of the phages KMM1–4, and the phage cocktail (4 × 10^8^ pfu/ml) for 6,12, 24, and 120 h. Bacterial 16S rRNA amplicon sequencing (V1-V2 region) was performed for six randomly selected polyps of each treatment and time point. **(A)** Box plots were used to depict alpha diversity via the Shannon (H′) index, comparing the microbiomes associated with *A. aurita* polyps affected by phage treatments with the untreated control polyps (CP). Significant changes in treatment were assessed using the Kruskal–Wallis and pairwise Wilcoxon test. Changes are highlights using asterisks at ^*^p < 0.05 and ^**^p < 0.01. **(B)** The bar plot illustrates the composition of untreated and phage-treated native *A. aurita* polyps after 6, 12, 24, and 120 h compared to untreated control polyps (before the experimental start). The data represents the median of six biological replicates. Bacterial genera with <0.1% relative abundance are grouped as “others,” and primary phage hosts are highlighted with*. **(C)** The PCoA visualizes weighted unifrac distances of phage-treated native polyps and untreated control polyps. Polygons group the six biological replicates per sample. Before analysis, ASVs with a relative abundance <0.01% in any sample were removed. The percentages of total inertia contributed by each axis are shown in the axis labels.

Shannon diversity analysis revealed no significant differences between polyps infected with phage KMM1 and the control (Figure 5A). However, exposure to KMM2, KMM3, KMM4, and the cocktail resulted in significantly divergent diversity between 12 and 24 h (Figure 5A), coinciding with the observation of substantial malformations (Figure 2A). Morphological patterns (Figure 2A) unveiled a consistent trend within the phage KMM2 and KMM3 treatment groups and within the KMM1 and KMM4 treatment groups. This consistency suggests a potential correlation between specific phage treatments and the resulting morphological outcomes in the polyps. The community changes were predominantly associated with the abundance shift of the Baltic Sea polyp’s main colonizer, uncultured *Mycoplasma* (ASV01479). Phage infection led to a decrease in *Mycoplasma* abundance by 29–69% during phenotype-affected time points (Figures 2B, 5B). In contrast, representatives of Bacteroidota and Proteobacteria experienced an increase in abundance. Within Bacteroidota, uncl. Flavobacteriaceae and *Polaribacter* exhibited noteworthy increases. For instance, after 6 h with presence of KMM1, uncl. Flavobacteriaceae increased by 20%, while KMM3, KMM4, and the cocktail treatments showed a lower increase of 12%. At 12 h, all treatments displayed an increase in uncl. Flavobacteriaceae, corresponding to the major effect on polyp malformation. Subsequently, a progressive decrease was observed at 24 h for all treatments, except the cocktail treatment, which exhibited a delayed effect. At 120 h, the relative abundance was lower compared to the control group, aligning with an increased presence of *Mycoplasma* and a recovery of malformed polyps. Variance over time in other Bacteroidota mirrored the trend of uncl. Flavobacteriaceae. Regarding Proteobacteria, the genera *Pseudoalteromonas*, *Pseudomonas*, *Vibrio*, uncl. Gammaproteobacteria, and *Thalassotalea* displayed an increase (Figure 5B). This trend was consistent across all treatments after 6 h, with a rise after 12 h for KMM3 and a lag for KMM4, reaching its peak after 24 h. A reduction of Proteobacteria at 120 h was observed for KMM1 and KMM2, while KMM3, KMM4, and the cocktail treatments showed a delayed decrease effect. This indicates that KMM3 and KMM4 had a more pronounced influence on the cocktail treatment compared to KMM1 and KMM2.

### 3.5 Phage infections cause fluctuations in the evenness of ASVs associated to polyp malformations

The different phages used differ in their host range, the primary target hosts of phage KMM1 were identified as *Pseudomonas* spp. and *Staphylococcus* spp., while the hosts for phages KMM2-KMM4 are classified to the *Citrobacter* genus. Thus, the changes in the microbiota was evaluated taking the primary host into account. In the KMM1 treatment, *Pseudomonas* persisted in the phage treatment with marginal changes in abundance, whereas *Staphylococcus* disappeared after 6 h. *Citrobacter* was absent in the KMM2 and KMM3 treatments after 6 h, and decreased with the KMM4 treatment. The cocktail treatment combined the effects of individual treatments. *Pseudomonas* marginally decreased in the beginning but increased again in abundance after 120 h, while *Staphylococcus* vanished after 6 h. *Citrobacter* initially increased and then vanished after 12 h (Figure 5B; Supplementary Table S4). In general, the treatment effects observed by analyzing the median of all biological replicates at the genus level were moderate and similar to the variations between different replicates (Figures 5B,C). Nevertheless, the PCoA validated the identified effects in alpha diversity, indicating shifts in the microbial community between 6 and 24 h, which recovered by 120 h (Figure 5C). Once more, these findings align with the outcomes observed in morphological monitoring (Figure 2). We hypothesize that the phage infection of the primary target hosts disrupts the equilibrium of the native polyp microbiome, explicitly impacting the evenness of ASVs, through changes in the main colonizer *Mycoplasma*. As a result, the polyp host exhibits malformations. Subsequently, the microbiome undergoes a re-balancing process, leading to the recovery of polyp fitness.

**Figure 6:**
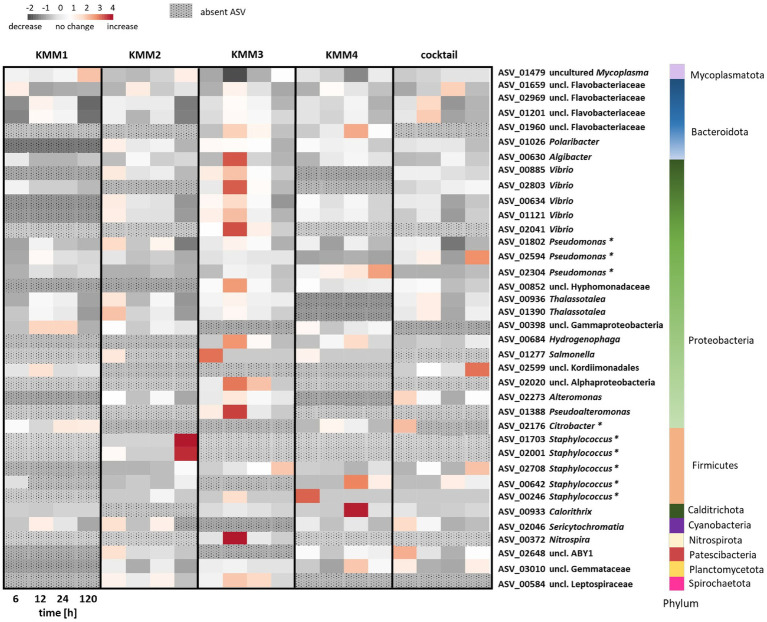
Changes in relative abundance of amplicon sequence variants (ASVs) exposed to different phage treatments. The interrelationships among ASVs in the microbiomes of polyps subjected to phages KMM1–4 and the phage cocktail for 6, 12, 24, and 120 h are depicted in a heatmap. Each column presents the median of six replicates, displaying each treatment’s relative abundances (>0.1%) at every time point. Heatmap values, representing changes in relative abundances, were normalized to control polyps. Rows correspond to 37 ASVs encompassing 23 genera from 10 different phyla, with particular notation (*) for primary phage hosts. The color scheme indicates changes in relative abundance compared to control conditions: dark grey signifies a decrease, white indicates no change, and red denotes an increase in abundance. ASVs that have disappeared in comparison to the control polyps are denoted by a dotted pattern.

To provide a more in-depth understanding and clarification of the observations made at the genus level, a detailed analysis of the impact of phages on individual ASVs was conducted. The analysis focused exclusively on the primary target hosts of phage infection and ASVs with a relative abundance >0.1%. Among the 2,682 ASVs considered, 37 ASVs exhibited alterations in relative abundance >0.1% compared to the control (Figure 6). The provided capacity on the polyp is utilized and balanced through shifts in abundance. In more detail, the phage KMM1, known for its broad host range, has been shown to infect *Pseudomonas* and *Staphylococcus*. Here, infection with KMM1 resulted in the depletion of *Staphylococcus* (ASV01703, ASV02001, ASV02708, ASV00642, ASV00246). However, *Pseudomonas* (ASV01802, ASV02594, ASV02304) decreased and then even slightly increased in abundance after 12–24 h. In addition, the main colonizer *Mycoplasma* (ASV01479) substantially decreased, allowing for an increase in the abundance of uncl. Flavobacteriaceae (ASV01659, ASV02969, ASV01201), uncl. Gammaproteobacteria (ASV00398), uncl. Kordimonadales (ASV02599), and *Sericytochromatia* (ASV02046) (Figure 6). Abundance shifts were identified between 6–24 h, aligning with significant changes in polyp fitness (Figures 2, 6). Following these shifts, the microbiome as well as the fitness of the polyp recovered, showing a decrease in the previously increased ASVs and the recovery of *Mycoplasma* (Figures 2, 6).

In a similar fashion, *Citrobacter* phages KMM2-4 lead to a consistent diminishment of *Citrobacter* in all these treatments. Nevertheless, distinct phages led to varied microbiome responses (Figure 6). KMM2 showed a reduction in *Mycoplasma* and an increase in *Vibrio*, *Thalassotalea*, *Pseudomonas*, and *Salmonella* relative abundances after 6 h (Figure 6). The microbiome re-balanced over 120 h, leading to a resurgence of *Mycoplasma* (Figure 6). Exposure to KMM3 once again indicated a decline in *Mycoplasma*, accompanied by an increase in uncl. Flavobacteriaceae, *Polaribacter*, *Algibacter*, *Vibrio*, *Pseudoalteromonas*, and other Proteobacteria, as well as *Nitrospira* (Figure 6). Shifts were observed between 6 and 24 h, coinciding with malformation observations (Figures 2, 6). Similarly, the re-balancing of the microbiome after 120 h, marked by the resurgence of *Mycoplasma*, contributed to the recovery of polyp fitness (Figures 2, 6). Infection with KMM4 demonstrated a less pronounced and delayed decrease in *Mycoplasma*, aligning with the observed malformations (Figures 2, 6). Particularly notable was the increase in uncl. Flavobacteriaceae (ASV01960), *Hydrogenophaga* (ASV00684), *Calorithrix* (ASV00933), and uncl. Gemmataceae (ASV03010) (Figure 6). Up to 120 h, *Mycoplasma* exhibited a gradual increase, coupled with the decrease of the previously mentioned ASVs during polyp fitness recovery (Figures 2, 6). Ultimately, exposure to the phage cocktail only partially resulted in combined effects of the individual phages, illustrating the intricacy of the microbiome response and potential interactions among phages. The cocktail exposure demonstrated a recoverable effect on *Mycoplasma* abundance (Figure 6). Additionally, the abundance of uncl. Flavobacteriaceae, *Thalassotalea*, *Alteromonas*, and *Sericytochromatia* were noted to act differently in the diverse treatments between 6–24 h (Figure 6). Notably, there was a high abundance of uncl. Kordiimonadales, likely not adversely affecting host fitness (Figures 2, 6).

Overall, the findings clearly show that phage treatments led to significant shifts in microbial community composition, particularly affecting the evenness of ASVs depicted as Shannon H′. These changes were observed in association with malformations in the polyps. We assume that phage infections disturbed the equilibrium of the native polyp microbiome, resulting in malformations. However, the microbiome exhibited a re-balancing process over time, contributing to the recovery of polyp fitness.

## 4 Discussion

Our investigation into the impact of phage infections on *A. aurita* Baltic Sea polyps and their microbiome has yielded intriguing findings. During a host-fitness experiment exposing polyps to isolated phages (BSwM KMM1-4) and a phage cocktail, we monitored survival rates, phenotypic changes, and microbiome alterations, offering comprehensive insights into phage-host-microbiome interactions (Figure 1).

### 4.1 Complex interactions among phages, microbiomes, and host organisms

The survival analysis indicated that sterile polyps, lacking their microbiota, were unaffected by phage infections, emphasizing the indirect nature of the observed phage effects on native polyps (Supplementary Figure S2). This implies that the alterations or responses observed in native polyps, such as morphological changes (Figure 2), are likely mediated by the interaction between phages and the associated microbiota in the native polyps. The microbiota may play a crucial role in modulating the impact of phage infections on the host organism, showcasing the intricate interplay between phages, microbiota, and the host’s survival and phenotype.

Intriguingly, exposure to KMM3, KMM4, and a phage cocktail induced notable changes in the generated native microbiome of *A. aurita* polyps, even in the absence of the host. The observed alterations encompassed shifts in the abundance of specific microbial taxa, suggesting that these phages possess the potential to influence the microbiome associated with *A. aurita* polyps. Notably, KMM4 exerted a substantial impact on the generated native microbiota, with *Pseudoalteromonas* and *Vibrio* emerging as the most abundant taxa (Figure 3A). This highlights KMM4’s pronounced effect on the microbiota composition in the absence of the polyp. However, the impact of KMM4 treatment appears to be mitigated and distinct in the presence of the polyp (Figure 5B). Of particular interest is the observed increase in the abundance of uncl. Flavobacteriaceae compared to other genera within the Protobacteria group. This nuanced interaction between KMM4 and the polyp’s microbiome prompts intriguing questions about the specific mechanisms at play, especially in the context of the polyp’s presence. Further investigation is warranted to elucidate the factors contributing to the observed mitigation of KMM4’s impact and the preferential increase in uncl. Flavobacteriaceae in the presence of the polyp. Understanding these dynamics could provide valuable insights into the complex interplay between phage treatments and the microbiome associated with *A. aurita* polyps.

The concept of interactions between phages, microbiota, and host organisms is an emerging research field (Mirzaei and Maurice, 2017; Chatterjee and Duerkop, 2018). A recent study on the human gut microbiota observed that bacteriophages can shape the composition of bacterial communities in the gut (Shkoporov et al., 2022). Changes in the abundance of specific phages were associated with alterations in the bacterial taxa present. This dynamic interplay between phages and bacteria in the gut microbiome can influence overall gut health and homeostasis (Shkoporov and Hill, 2019; Li et al., 2021). Research in human individuals with cystic fibrosis (*CF*) investigated the impact of bacteriophages on the lung microbiome (Kiedrowski and Bomberger, 2018; Ling et al., 2023). Phage-bacterial interactions contributed to the dynamics of bacterial populations in the *CF* lung, potentially influencing disease progression (Megremis et al., 2023). Understanding these interactions might have implications for developing phage-based therapies, for instance for such respiratory infections. Studies on marine environments have also explored the role of bacteriophages in shaping microbial communities (Breitbart et al., 2008; Rohwer and Thurber, 2009; Coutinho et al., 2017; Breitbart et al., 2018). The impact of phages on ocean bacterial populations can have cascading effects on nutrient cycling and ecosystem dynamics (Breitbart et al., 2008; Mayers et al., 2023). This extends to the host organisms within marine ecosystems, highlighting the interconnected nature of phage-microbiome-host interactions.

### 4.2 Dynamic adaptability in *Aurelia aurita*: host responses to environmental disturbances such as phages

The survival rates of native polyps following phage infections remained unaffected, with only a small percentage displaying morphological abnormalities (Figure 2), implying a nuanced interplay between the host organism, its microbiota, and the phages. This resilience of native polyps to phage-induced disturbances may be attributed to inherent mechanisms or adaptations in native polyps that mitigate the impact of specific bacteriophage infections, possibly involving immune responses, biochemical defenses, or other factors (Barr et al., 2013; Kronheim et al., 2018; Popescu et al., 2021). While some individuals exhibit abnormalities, the overall fitness and survival rates of native polyps are not significantly compromised.

The observed changes in polyp morphology and microbiota dynamics can be linked to the introduction of non-native phages into the metaorganism. This parallels findings from studies where polyps were exposed to potentially pathogenic bacteria (Weiland-Bräuer et al., 2020a,b), resulting in the modulation of the native microbiota. The presence of phages, with their ability to infect and eliminate specific bacteria, led to alterations in the composition and abundance of the microbiota associated with *A. aurita* polyps. The interaction between bacteriophages and bacteria could indirectly influence the health and morphology of *A. aurita* polyps. Notably, changes in the abundance of specific bacteria due to phage introduction, such as *Mycoplasma*, Bacteroidota, and Proteobacteria, might impact the polyp phenotype if these bacteria are integral to maintaining polyp health.

Compensatory mechanisms, such as repair processes, stress responses, or microbiome-mediated protection, could minimize the negative effects of phages (Barr et al., 2013; Koskella et al., 2017; Fernández et al., 2018). The transient malformations observed in up to 10% of native polyps, which demonstrate the resilience of *A. aurita* to phage-induced disturbances, can be explained by multiple factors. Firstly, the low proportion of malformations suggests the overall population’s fitness remains robust, with most individuals successfully recovering from phage-induced disturbances (Wendling et al., 2017; Podlacha et al., 2021). *A. aurita’s* high regenerative capacity and ecological resilience contribute to this adaptability, enabling swift recovery from disturbances (Lucas, 2001; Mitchell et al., 2021; Pinnow et al., 2023). Secondly, the malformations may be a temporary stress response, reversible once the phage-induced stressor is removed or mitigated (Lucas and Horton, 2014; Conley and Uye, 2015; Pinnow et al., 2023). Thirdly, *A. aurita’s* adaptive plasticity allows it to modify its morphology in response to environmental changes, including phage disturbances, with the transient nature of malformations indicating a quick reversion to the normal state (Lucas and Horton, 2014). The observed resilience is hypothesized to be linked to the dynamic nature of *A. aurita’s* microbiome, where transient malformations arise from shifts in microbial community structure, and recovery aligns with the re-establishment of a balanced microbiome composition.

### 4.3 Phage-driven microbiome changes in *Aurelia aurita*

Microbiome analysis through 16S rRNA amplicon sequencing provided a comprehensive view of phage-induced changes. The experiment demonstrated that phage infections, particularly with KMM3, KMM4, and the phage cocktail, caused significant shifts in the polyp microbiome composition (Figure 5).

Moreover, the morphological patterns of malformed polyps displayed a consistent trend among the treatment groups of phages KMM2 and KMM3, as well as between KMM1 and KMM4. This intriguing observation suggests a potential correlation between specific phage treatments and the morphological appearance of polyps. To delve deeper into the underlying factors influencing polyp morphology in response to phage introduction, several possibilities can be considered. The infectious potential of each phage (Stante et al., 2023), being yet unknown, necessitates further analyses, including a comprehensive assessment of their impact on low-abundant bacteria and uncultured species. Bioinformatics tools are proposed for analyzing phage genomes, supplementing traditional laboratory assays. Additionally, the rapid adaptation of bacteria to specific phages, as demonstrated in other systems such as the human gut (Seed et al., 2014), could contribute to the observed morphological variations in *A. aurita* polyps. Furthermore, the different reactions of polyps following phage challenge might be linked to defense mechanisms of the bacterial community (i.e., Restriction-Modification systems, CRISPR systems, antibacterial proteins, biofilm formation, Quorum Sensing) (Wilson and Murray, 1991; Horn et al., 2016; Miernikiewicz et al., 2016; Fernández et al., 2018; Zhou et al., 2023) or *A. aurita* polyps (e.g., innate immunity, humoral immune responses, cellular immune responses) (Ochs et al., 1992; Gorski et al., 2003; Krut and Bekeredjian-Ding, 2018; Carroll-Portillo and Lin, 2019), pointing to the intricate interplay between phage treatments and host immune dynamics. As the understanding of these dynamics evolves, alternative methods and sequencing technologies are recommended for a more comprehensive exploration. This includes investigating potential delayed or cumulative effects on the microbiome and unraveling the intricate and interconnected nature of microbial communities in response to phage challenges.

Alpha diversity analyses showed alterations in ASV evenness, especially during the critical 12–24 h timeframe corresponding to observed phenotypic changes in the polyps. This underlines the intricate relationship between the host’s phenotype and its associated microbial community, suggesting a bidirectional interaction wherein changes in the microbiome influence the host’s phenotype and vice versa. In animal microbiomes, bacteriophages are recognized as integral components that actively shape microbial community composition and dynamics (Breitbart et al., 2008; Koskella and Meaden, 2013; Koskella and Brockhurst, 2014; Fernández et al., 2018). They can influence the abundance and diversity of bacteria within an animal host by infecting and targeting specific bacterial species, directly impacting the microbial composition (Koskella and Meaden, 2013; Federici et al., 2021). Modulating microbial communities by bacteriophages in animal hosts can significantly affect host health (Peduzzi et al., 2014; Chatterjee and Duerkop, 2018; Vandamme and Mortelmans, 2019). The overall health of animal hosts heavily relies on sustaining a well-balanced microbial community (Weiland-Bräuer et al., 2015, 2020a,b), and natural bacteriophages can actively contribute to this equilibrium, playing a role in preserving the host’s microbiome homeostasis (De Paepe et al., 2014). Examining phage interactions with primary target hosts showed such intricate dynamics (Figure 6). *Staphylococcus*, for instance, was no longer detectable in response to phage infections, demonstrating species-specific susceptibility. Generally, the primary phage target hosts are low in abundance in the microbiome of *A. aurita*. The targeted elimination of these low-abundant members can lead to shifts in overall microbial composition (Figures 5, 6), creating ecological niches exploitable by other microorganisms influencing relative abundance (Sadiq et al., 2021). Complex bacterial interactions within the community may be disrupted, and even the removal of minor players can cascade through the network of interactions (Dawson et al., 2017). Members with a low-abundant may contribute unique functions or metabolic pathways to the microbiome (Cena et al., 2021; Foo et al., 2021). Their removal can result in the loss or alteration of these functions, affecting the overall functional potential. In addition, they may also play a role in stabilizing the microbiome (Benjamino et al., 2018). Their removal could, thus, destabilize the community, increasing susceptibility to fluctuations or invasion by opportunistic species. Furthermore, the remaining microbiome members may exhibit resilience and adaptability in response to removing low-abundant taxa (Hadaidi et al., 2017; Doering et al., 2021), involving changes in community structure or the development of resistance mechanisms. The intricate and interconnected nature of microbial communities makes it challenging to predict all consequences accurately. Long-term studies are essential to capture potential delayed or cumulative effects on the microbiome.

### 4.4 Phage competition dynamics influencing species-specific interactions in *Aurelia aurita*

We further observed potential phage competition, especially in the phage cocktail treatments (Figures 5, 6), which have been shown to shape the complex microbiome landscape (Abedon, 2009; Diaz-Munoz, 2017). Diverse phages with varied host ranges selectively target bacterial species in these scenarios, creating intricate species-specific interactions (Holmfeldt et al., 2007; Stone et al., 2019). Phages exert selective pressure, favoring resistant bacterial species and leading to dynamic microbiome reconfiguration (Labrie et al., 2010; Yeoman et al., 2011). Competitive exclusion mechanisms play a role in influencing resource competition and niche occupancy (García-Bayona and Comstock, 2018). Microbial communities show elasticity and adaptability, which reach a new equilibrium in light of fluctuations. Furthermore, changes in the abundance of specific genera can impact the microbiome’s overall metabolic capabilities and functional diversity (Jiang et al., 2016; Lesser et al., 2022).

### 4.5 Importance of *Aurelia aurita*-specific *Mycoplasma* in phage-host interactions

While phages exhibited host specificity, impacting bacterial genera such as *Citrobacter* or *Pseudomonas/Staphylococcus*, the most substantial effect in response to the phages was observed in the abundance of the uncultured, potentially novel *A. aurita*-specific *Mycoplasma* (Figures 4–6). However, sequence analysis revealed pronounced variability among replicates, which may have obscured subtle changes in *Mycoplasma* abundance, indicating a limitation of our analysis. Nevertheless, the dynamic behavior of *Mycoplasma* and its central role in the microbiome hint at its potential importance for *A. aurita’s* overall health. *Mycoplasma*, a genus characterized by its small size and absence of a cell wall, is known for engaging in symbiotic relationships within marine hosts (Razin, 1978; McCutcheon and Moran, 2012; Stabili et al., 2020). These relationships vary from mutualistic to commensal, influencing host health and contributing to ecological balance (Casadevall and Pirofski, 2000; Mushegian and Ebert, 2016). For instance, *Mycoplasma mobile* has been identified in the gills of rainbow trout, suggesting a potential connection with the fish’s respiratory system (Stadtländer et al., 1995). Additionally, marine invertebrates like sea anemones and corals have been found to harbor *Mycoplasma*-related microbes, impacting nutrient cycling, symbiosis, and coral health (Neulinger et al., 2009; Murray et al., 2016; Woo et al., 2017). Identifying *A. aurita*-specific *Mycoplasma* in the microbiome of Baltic Sea polyps raises further questions about its potential symbiotic role in supporting the host’s well-being. In a study in 2015, we detected a likely novel *Mycoplasma* strain in Baltic Sea medusae, potentially residing within the epithelium based on FISH analysis, suggesting that is an endosymbiotic symbiont. The identified *Mycoplasma* sequences exhibited 84% similarity to an uncultured bacterial clone, “CFI73,” derived from the digestive tract of *Octopus mimus* (Iehata et al., 2015). This clone is closely related to members of the orders Entomoplasmatales and Spiroplasmatales. These two orders were also identified as the closest neighbors in the phylogenetic tree (Supplementary Figure S3) to our uncultured *Mycoplasma* from the *A. aurita* polyps. This finding confirms that, in addition to the medusae from the Baltic Sea in 2015 (Weiland-Bräuer et al., 2015), also polyps kept in captivity are inhabited to a large extent by the uncultured *Mycoplasma* member. The presence of uncultured *Mycoplasma* in pelagic medusae and benthic polyps underlines the potential specificity of this bacterium for the Baltic Sea subpopulation of *A. aurita*. There it is likely to contribute substantially to the well-being of its eukaryotic host. Investigating this *Mycoplasma’s* genetic and functional features can unveil the distinct ways it contributes to the microbiome of *A. aurita* and the metaorganism, shedding light on its ecological adaptability. Despite the potential importance of the *A. aurita*-specific *Mycoplasma*, working with this genus is experimentally challenging. These bacteria often represent endosymbionts, are known for their small genome sizes and limited metabolic capabilities, making them difficult to culture and study in laboratories (Razin, 1978; Bölske, 1988). The absence of a cell wall adds complexity, affecting antibiotic susceptibility and requiring specialized cultivation techniques (Stanbridge, 1971; Phillips, 1978; Razin, 1978). Moreover, *Mycoplasma’s* fastidious growth requirements necessitate creating conditions that mimic its natural environment, further complicating experimental setups (Stanbridge, 1971; Razin, 1978; Wisselink et al., 2019). The interaction between phages and *A. aurita’s* microbiome unveils the central role of *Mycoplasma*. Understanding *Mycoplasma’s* symbiotic roles in the marine environment, its potential novelty and the challenges associated with working with these bacteria contribute to a more comprehensive exploration of its significance to *A. aurita’s* health. Overcoming the challenges in studying *Mycoplasma* and researching its specific functions in the *A. aurita* host, will enhance our understanding of the intricate relationships within marine microbiomes.

## 5 Conclusion

Overall our findings contribute to the broader understanding of phage-host-microbiome interactions, providing a foundation for continued investigations into the ecological dynamics of marine environments. This knowledge is not only valuable for comprehending the delicate balance within the *A. aurita* ecosystem, but also has implications for broader marine microbiome research. Additionally, this in-depth knowledge can potentially direct management strategies for marine health and resilience in the face of environmental challenges.

## Data availability statement

Original datasets are available in a publicly accessible online repository. Data of 16S rRNA genes amplicon sequencing were deposited under the NCBI BioProject PRJNA1010146, and BioSample Accessions SAMN37180193-SAMN37180465. Sequence data of uncultured Mycoplasma clone 1 was deposited under the GenBank Accession no. OR634772.

## Author contributions

MS: Data curation, Formal analysis, Investigation, Methodology, Validation, Visualization, Writing – original draft, Writing – review & editing. NW-B: Conceptualization, Supervision, Validation, Visualization, Writing – original draft, Writing – review & editing. AH-H: Data curation, Formal analysis, Software, Validation, Visualization, Writing – original draft, Writing – review & editing. RS: Conceptualization, Funding acquisition, Project administration, Resources, Supervision, Writing – original draft, Writing – review & editing.

## Footnotes

^1^ https://biit.cs.ut.ee/clustvis/

^2^ https://imedea.uib-csic.es/mmg/ltp/
