# Supplementary material for: Marine bacteriophages disturb the associated microbiota of Aurelia aurita with a recoverable effect on host morphology
Source: Front Microbiol. 2024 Mar 11;15:1356337. doi: 10.3389/fmicb.2024.1356337 (PMC10964490; doi:10.3389/fmicb.2024.1356337)
Supplement: Supplementary file 1 [file Data_Sheet_1.ZIP › Supplementary_Material_Stante et al/Supplementary_Material_Stante et al..docx]

Supplementary Material

Marine bacteriophages disturb the associated microbiota of *Aurelia aurita* with a recoverable effect on host morphology

M. Stante^1^, N. Weiland-Bräuer^1^, A. von Hoyningen-Huene^1^, R. A. Schmitz^1,^ *

^1^ Institute for General Microbiology, Christian Albrechts University, Am Botanischen Garten 1-9, D-24118 Kiel, Germany;

* Correspondence: Ruth Anne Schmitz: rschmitz@ifam.uni-kiel.de

# Supplementary Figures and Tables

**Table S1: Statistical analyses of host-fitness parameter malformation of *A. aurita* Baltic Sea polyps.** A significance cut-off of p ≤ 0.05 was employed in a proportionality test (specifically, a 2-proportions test without 'Yates' continuity correction) using the stats package version 4.2.3 in R, assessing significant differences between the proportion of healthy polyps and harmed polyps.

**Table S2: Bacterial amplicon sequencing variants (ASVs).** After denoising, ASV filtering, and contaminant removal, 2,682 identified ASVs are listed with their taxonomic assignment and corresponding relative abundances in the various treatments.

**Table S3: Metadata and calculated diversity and richness indices for all samples in the host-fitness experiment.** Diversity and richness indices were calculated on samples which were rarefied at 10,000 reads. Faith’s phylogenetic diversity was calculated using a midpoint-rooted phylogenetic tree of the representative sequences and the rarefied reads. Samples NTC and MOCK represent the sequencing negative and positive controls.

**Table S4: Relative abundance of primary phage target hosts.** Relative abundances of primary target bacteria (Pseudomonas, Citrobacter, and Staphylococcus) are shown, including untreated native polyps at 0 h (control) and the phage-treated native polyps at 6, 12, 24, and 120 h.

# Supplementary Figures


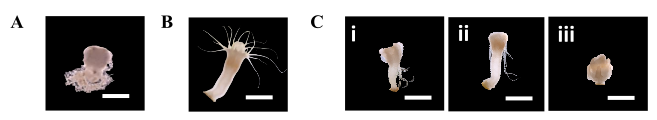


**Figure S1. Classification of polyp phenotypes.** Tentacle presence, phenotypic appearance, and proportions were used to classify the polyps' fitness into two categories: unaffected and malformed. Scale bars represent 1 mm. (A) Dead polyp. (B) Extended, completely grown tentacles, an expanded calyx, and a stalk attachment present healthy, unaffected polyp. (C) Malformed polyps lost two or more characteristics, encompassing a deformed calyx (Ci) and reduced, absorbed (Cii), or even absent (Ciii) tentacles.


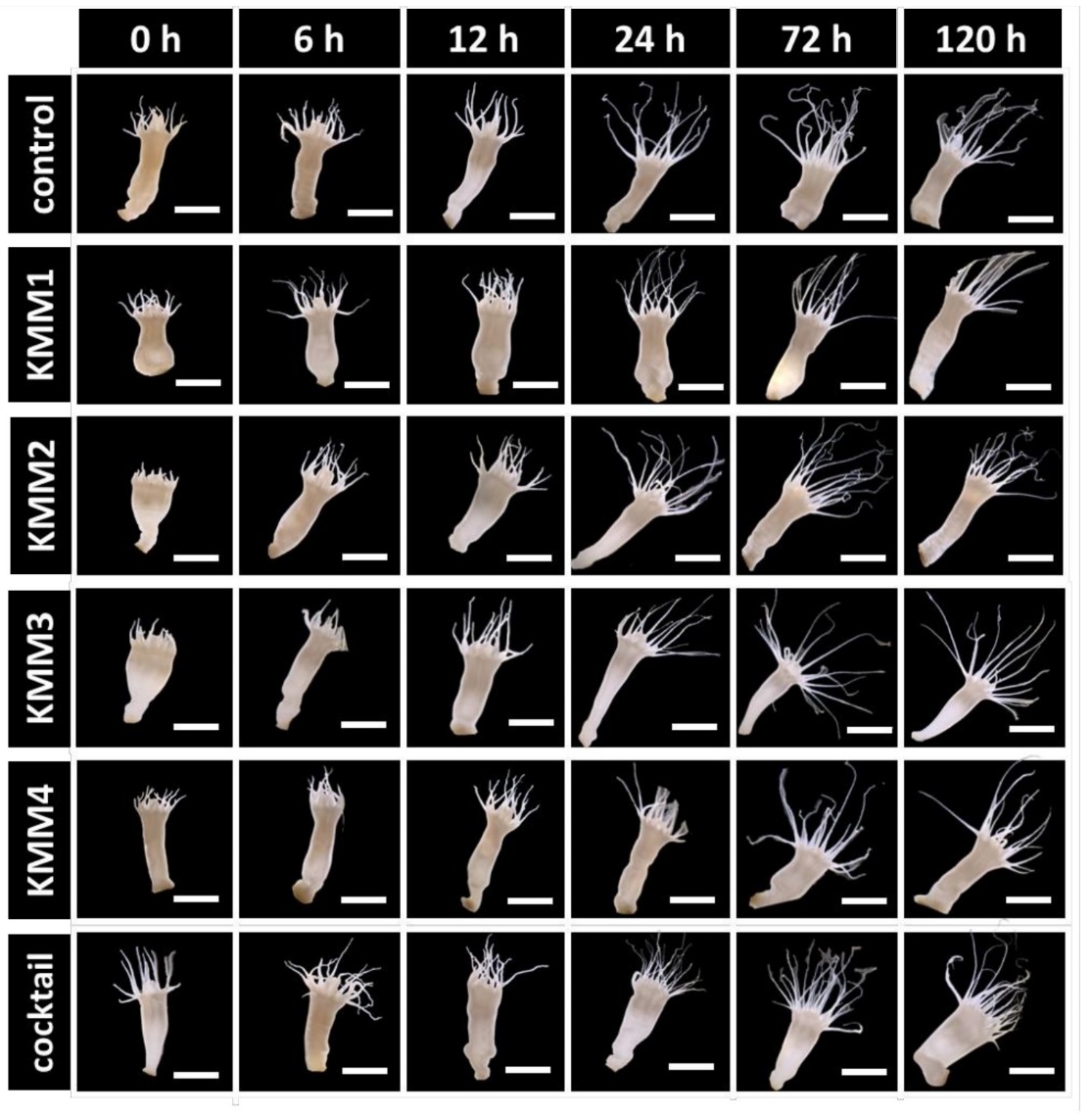


**Figure S2: Impact of bacteriophages on the phenotypical appearance of sterile *A. aurita* polyps.** Sterile *A. aurita* polyps were generated and kept in 1 ml of sterile ambient water. Polyps (48 replicates) were individually exposed to 10 µl of one of the phages KMM1 - 4 (1 x 10^8^ pfu/ml) and the phage cocktail (4 x 10^8^ pfu/ml) for up to 120 h. Polyp morphology was monitored (Novex Binocular RZB-PL Zoom-Microscope 65.500, Arnhem, Netherlands). Representative original photographs display no discernible phenotypic alterations through the treatment and over time. Scale bars represent 1 mm.


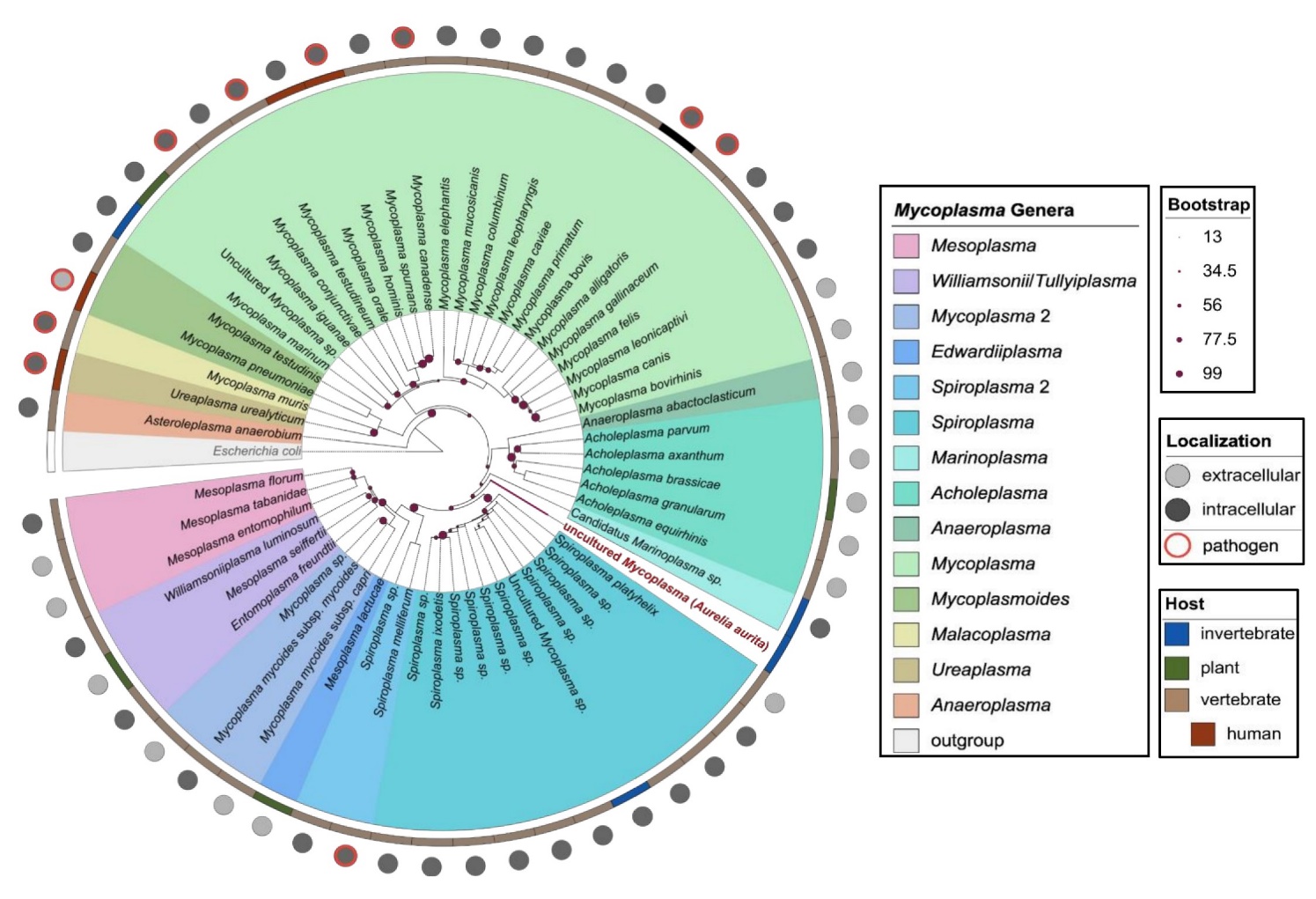


**Figure S3: Taxonomic classification of the highly abundant *A. aurita*-specific uncultured *Mycoplasma*.** The phylogenetic tree is based on a subset of the "All Species Living Tree” (LTP) where *Mycoplasma* sequences of interest where integrated. The resulting phylogenetic tree was visualized using iTOL v6, including the selected *Mycoplasma* representatives' potential host origin (colored bars), localization (circles), and pathogen status (circle outlines). The *A. aurita*-specific *Mycoplasma* branches off from known *Spiroplasma* and *Mesoplasma* representatives.
