# Supplementary figures and images for: Marine bacteriophages disturb the associated microbiota of Aurelia aurita with a recoverable effect on host morphology
Source: Front Microbiol. 2024 Mar 11;15:1356337. doi: 10.3389/fmicb.2024.1356337 (PMC10964490; doi:10.3389/fmicb.2024.1356337)

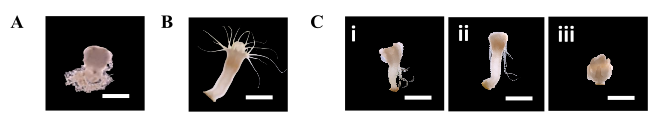

Supplement: Supplementary file 1 [file Data_Sheet_1.ZIP › Supplementary_Material_Stante et al/Supplementary Figure1_Stante et al..tiff]

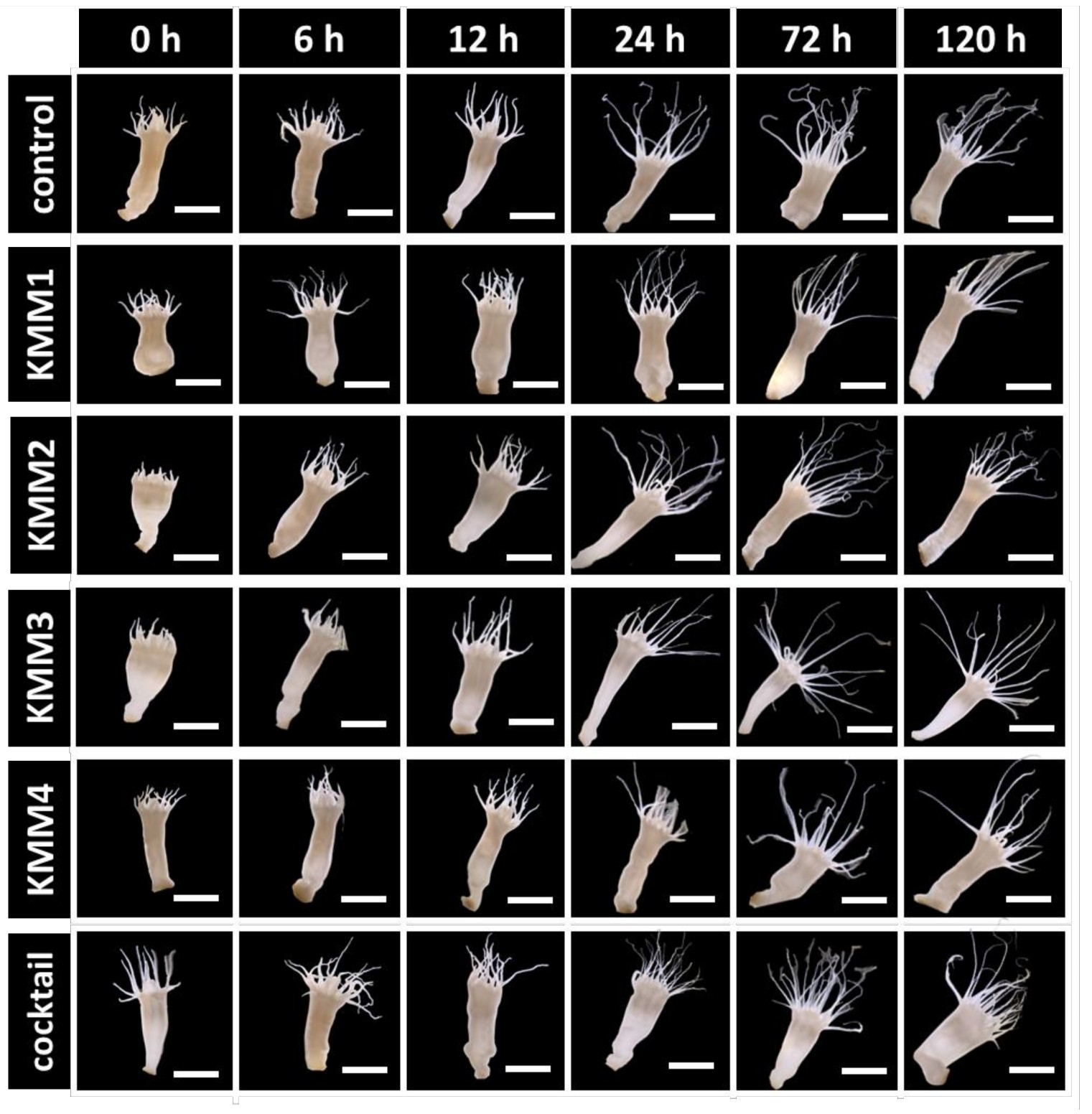

Supplement: Supplementary file 1 [file Data_Sheet_1.ZIP › Supplementary_Material_Stante et al/Supplementary Figure2_Stante et al...jpg]

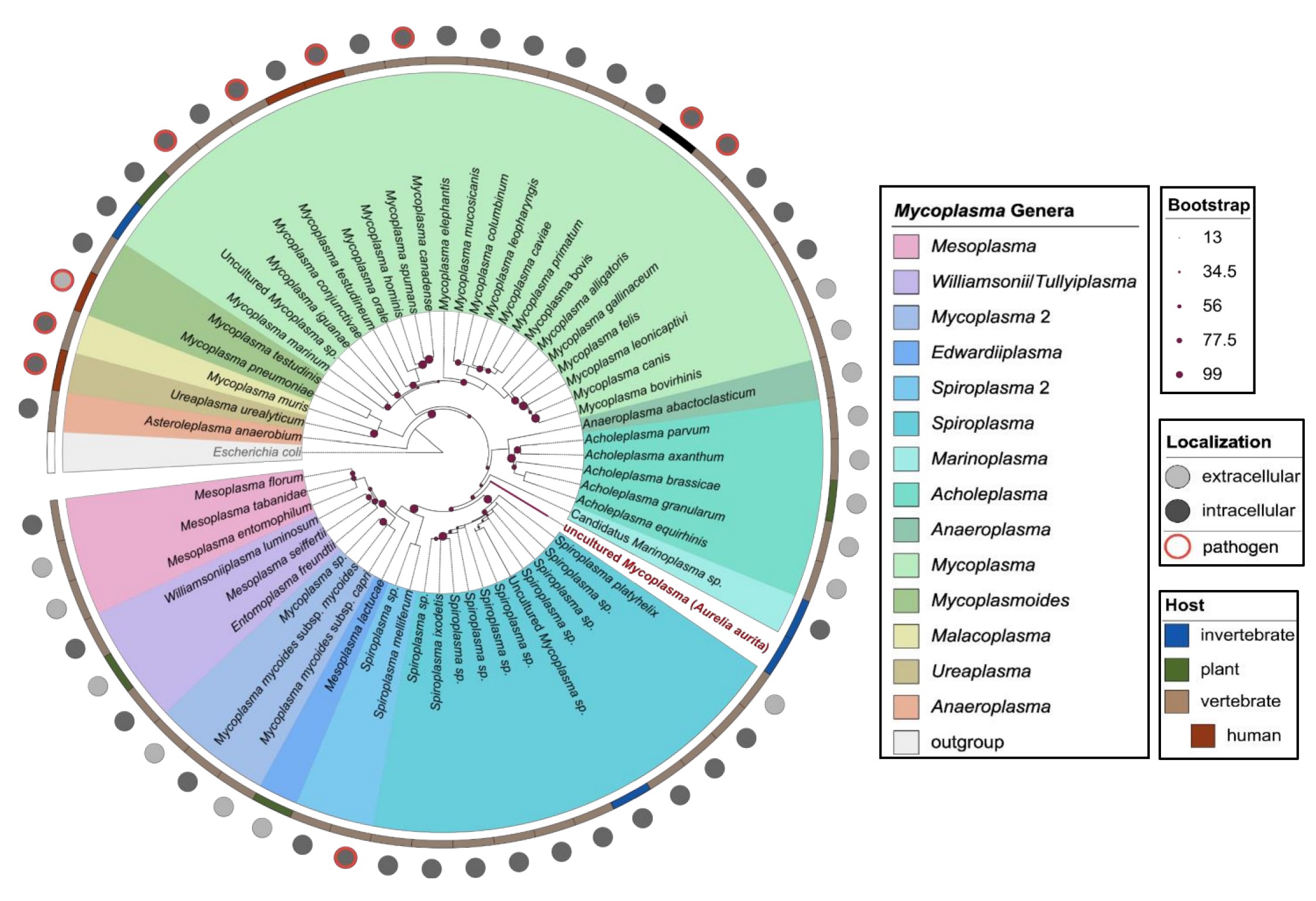

Supplement: Supplementary file 1 [file Data_Sheet_1.ZIP › Supplementary_Material_Stante et al/Supplementary Figure3_Stante et al..jpg]
